# Phenylalanine *meta*‐Hydroxylase: A Single Residue Mediates Mechanistic Control of Aromatic Amino Acid Hydroxylation

**DOI:** 10.1002/cbic.201900320

**Published:** 2019-07-18

**Authors:** Sabine Grüschow, Joanna C. Sadler, Peter J. Sharratt, Rebecca J. M. Goss

**Affiliations:** ^1^ School of Chemistry University of St. Andrews North Haugh St. Andrews KY16 9ST UK; ^2^ Department of Biochemistry University of Cambridge Tennis Court Road Cambridge CB2 1GA UK

**Keywords:** biocatalysis, enzyme mechanisms, hydroxylases, *meta*-tyrosine biosynthesis, 1,2-hydride (NIH) shift

## Abstract

The rare nonproteinogenic amino acid, *meta*‐l‐tyrosine is biosynthetically intriguing. Whilst the biogenesis of tyrosine from phenylalanine is well characterised, the mechanistic basis for *meta*‐hydroxylation is unknown. Herein, we report the analysis of 3‐hydroxylase (Phe3H) from *Streptomyces coeruleorubidus*. Insights from kinetic analyses of the wild‐type enzyme and key mutants as well as of the biocatalytic conversion of synthetic isotopically labelled substrates and fluorinated substrate analogues advance understanding of the process by which *meta*‐hydroxylation is mediated, revealing T202 to play an important role. In the case of the WT enzyme, a deuterium label at the 3‐position is lost, whereas in in the T202A mutant 75 % retention is observed, with loss of stereospecificity. These data suggest that one of two possible mechanisms is at play; direct, enzyme‐catalysed deprotonation following electrophilic aromatic substitution or stereospecific loss of one proton after a 1,2‐hydride shift. Furthermore, our kinetic parameters for Phe3H show efficient regiospecific generation of *meta*‐l‐tyrosine from phenylalanine and demonstrate the enzyme's ability to regiospecifically hydroxylate unnatural fluorinated substrates.

## Graphical abstract

**The final biosynthetic step** to *meta*‐tyrosine, an unnatural amino acid found in biologically active natural products, is mediated by phenylalanine *meta*‐hydroxylase. An integrated kinetics analysis, isotope labelling and mutational analysis study identified T202 as mediating mechanistic control of this intriguing enzyme. The synthetic utility of this enzyme was expanded to fluorinated phenylalanine analogues.

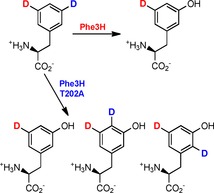

## Introduction

**Scheme 1:**
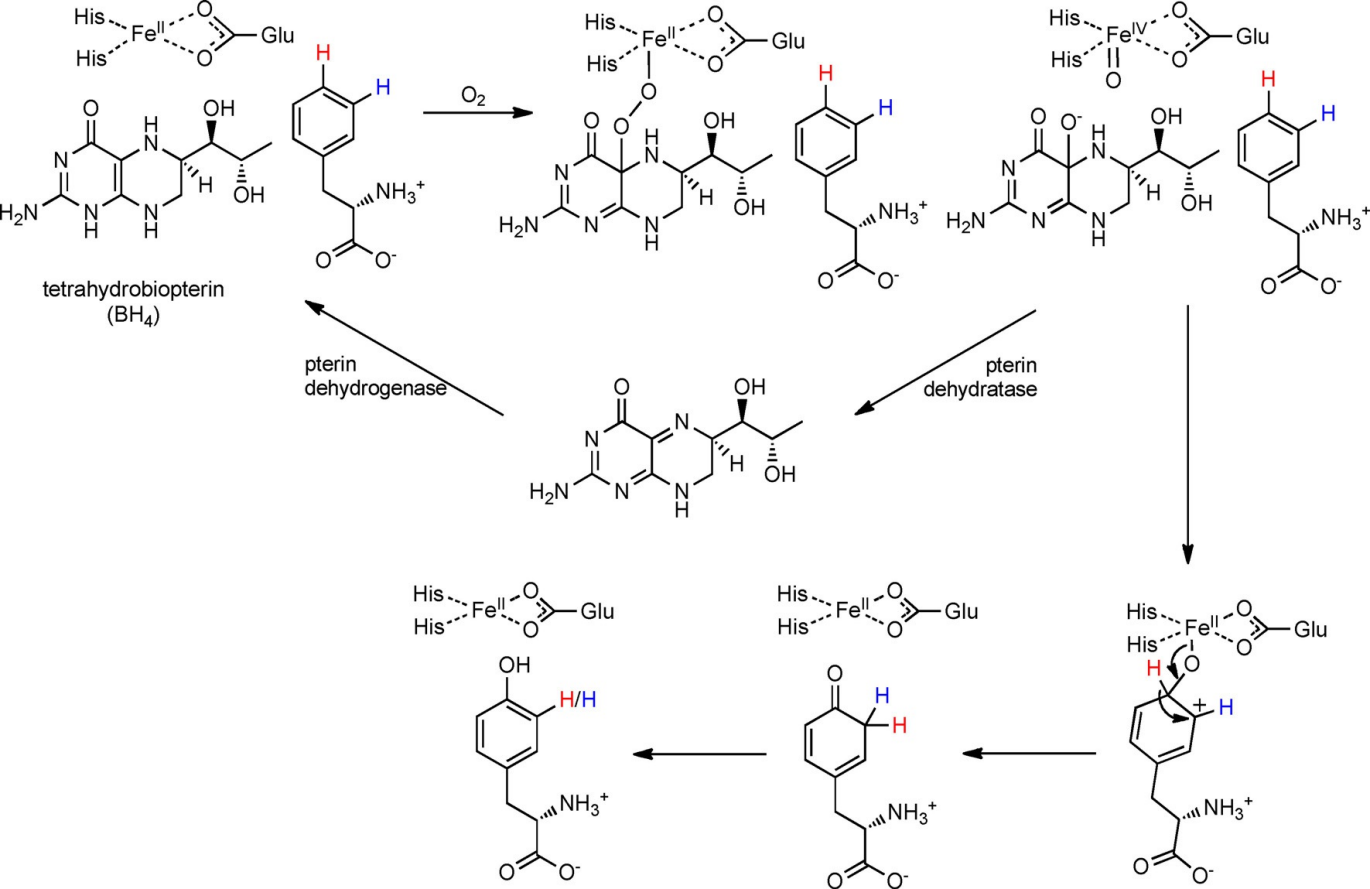
The established mechanism of tyrosine biogenesis from phenylalanine mediated by a mammalian non‐heme‐iron hydroxylase and using the cofactor tetrahydrobiopterin (BH_4_) to yield the *para*‐hydroxylated product.

Aromatic amino acid hydroxylases represent a small but important group of enzymes responsible for a series of metabolically essential transformations, including the hydroxylation of l‐tyrosine, l‐tryptophan or l‐phenylalanine to yield l‐DOPA, 5‐hydroxy‐l‐tryptophan or l‐tyrosine, respectively.^1^ These non‐heme‐iron‐dependent enzymes bind their metal cofactor at the active site with one glutamate and two histidine residues, a binding mode that is also found in other non‐heme‐iron enzymes such as the α‐ketoglutarate‐dependent dioxygenases.^2^, ^3^ A significant difference from other non‐heme‐iron‐dependent enzymes is the requirement for tetrahydropterin as an additional cofactor. The mammalian aromatic amino acid hydroxylases have been extensively studied; this revealed a common catalytic cycle (Scheme 1).^4^, ^5^, ^6^ The cycle starts with the ferrous iron enzyme complex binding the tetrahydropterin cofactor, the amino acid substrate and molecular oxygen. The latter then reacts with the tetrahydropterin to form a μ‐bridged pterin–iron intermediate, which has been proposed to be the precursor of the active ferryl‐oxo species. This species serves as the electrophile for an electrophilic aromatic substitution reaction with the phenylalanine substrate through a cationic intermediate, which then undergoes 1,2‐hydride (NIH) shift and tautomerisation to yield the hydroxylated amino acid. The ferrous state of the iron is re‐established, and the product and hydroxypterin (4a‐carbinolamine) are released from the enzyme. Additional enzymes, pterin dehydratase and pterin dehydrogenase, recycle the pterin co‐factor.^4^, ^7^, ^8^ Whilst aromatic amino acid hydroxylation is essential in mammalian systems, prokaryotes synthesise aromatic amino acids according to the Shikimate pathway and, as such, hydroxylases are less abundant in bacteria.^9^ An interesting member of the bacterial hydroxylase family that has not been found in eukaryotes is phenylalanine 3‐hydroxylase (Phe3H), which catalyses the biogenesis of *meta*‐tyrosine (*meta*‐Tyr) from phenylalanine.^10^ *meta*‐Tyr is found in a small number of bacterial natural products, including the immunosuppressant sanglifehrin, thaxtomin (a metabolite associated with the appearance of potato scab) and the uridyl peptide antibiotics (UPAs) such as the pacidamycins and structurally related napsamycins, mureidomycins and sansanmycins. Within the UPA family, the presence of *meta*‐Tyr enables the formation of bicyclic phenylalanine derivatives through a Pictet Spengler reaction with a small series of aldehydes, thereby masking the terminal amine, which is known to be critical for antibiotic activity and plausibly conferring resistance and host protection.^11^, ^12^

Inactivation of the gene encoding phenylalanine 3‐hydroxylase (Phe3H) in *Streptomyces coeruleorubidus* results in the loss of production of *meta*‐Tyr‐containing pacidamycins, thus demonstrating the essential nature of the gene for *meta*‐Tyr production.^13^ *meta*‐Tyr also has a level of herbicidal activity and is used by certain grasses in allelopathy, where it is thought be synthesised by an unidentified P450 enzyme.^14^, ^15^ In mammals, *meta*‐Tyr is generated nonenzymatically from phenylalanine under conditions of oxidative stress.^16^, ^17^

Phe3H from *S. coeruleorubidus* shares 75–78 % amino acid sequence identity with proteins encoded in the biosynthetic gene clusters for mureidomycin, napsamycin and sansanmycin and the cluster for the structurally unrelated sanglifehrin (51 % identity at the protein level). The *meta*‐Tyr moiety in thaxtomin, on the other hand, is likely to be derived from a cytochrome P450‐catalysed tailoring step.^18^ To date, none of these hydroxylases has been studied in detail, and little is known about their catalytic mechanism.^10^ In comparison, the sequence identity of phenylalanine 3‐hydroxylases to characterised bacterial phenylalanine 4‐hydroxylases ranges from 24–28 %.

Walsh and co‐workers have previously reported kinetic parameters for Phe3H from *S. coeruleorubidus* and carried out preliminary mutational analysis of putative active‐site residues.^10^ However, to date it is unknown if Phe3H operates by a mechanism analogous to that for Phe4H (Scheme 1) or by another mechanism. We have furthered our understanding of this intriguing enzyme with additional analysis of its Michaelis–Menten kinetics and identification of inhibitors, and show its ability to regiospecifically hydroxylate a series of fluorinated substrates. These data prompted us to perform a series of isotope labelling and mutational analysis experiments, which revealed that, unlike other characterised amino acid hydroxylases, the electrophilic aromatic substitution may proceed with enzyme‐catalysed deprotonation prior to the tautomerisation step and does not necessarily involve an NIH shift, with this mechanistic control being mediated by the T202 residue.

## Results and Discussion

### Kinetics of Phe3H

The steady‐state kinetic parameters for Phe3H with two different pterin cofactors were determined (Table 1 and Figure S7 in the Supporting Information) under optimised assay conditions (Figure S2). In comparison to the previously reported data, which were obtained at pH 6, we observed a 30‐fold increase in *k*_cat_ and twofold increase in *K*_M_ working at pH 8.^10^ The intricate interplay between the iron, pterin and substrate is highlighted by entries 1 and 2, in which the pterin cofactor is DMPH_4_ or (±)‐6‐methyl‐5,6,7,8‐tetrahydropterin (6MPH_4_), respectively. The only structural difference between these pterins is an additional methyl group in DMPH_4_. Using DMPH_4_ rather than 6MPH_4_ results in an approximately 50 % increase in *K*_M_ for l‐Phe and, conversely, an almost twofold increase in *k*_cat_. Whereas substrate inhibition was observed when 6MPH_4_ was used as a cofactor (Table 1, entry 3), it was not observed with DMPH_4_. To enable better comparison with literature data, 6MPH_4_ was selected for further analysis of Phe3H.^10^

**Table 1:** Kinetic parameters for Phe3H for different substrates and cofactors.

	Substrate	Cofactor	*K*_M_^[a]^	*k*_cat_	*k*_cat_/*K*_M_
			[mm]	[min^−1^]^[a]^	[mm ^−1^ min^−1^]
1	l‐Phe	DMPH_4_	0.65±0.04	75±1	120±2.0
			(0.098±0.01)	(78±3)	(800±39)
2	l‐Phe	6MPH_4_	0.40±0.06	38±2	95±7.1
			(0.039±0.002)	(48±2)	(1200±55)
			(0.11±0.02)^[c]^	(89±9)^[c]^	(840±110)
3^[b]^	l‐Phe^[b]^	6MPH_4_^[b]^	1.1±0.2	1.3±0.1	1.2±0.13
			(0.026±0.004)^[b]^	(1.2±0.04)^[b]^	(46.16±2.6)
4	4F‐dl‐Phe	6MPH_4_	0.23±0.06	14±0.5	63±6.4
5	2F‐dl‐Phe	6MPH_4_	1.9±0.1	10±0.2	5.3±0.12
6	[3,5‐D_2_]‐l‐Phe	6MPH_4_	0.54±0.07	34±1	63±2.9

[a] Michaelis–Menten kinetic parameters for the amino acid substrate with fixed pterin concentration; values for the pterin cofactor with fixed l‐Phe concentration are provided in brackets. [b] Kinetic parameters reported by Zhang et al. [c] Michaelis–Menten parameters with substrate inhibition, *K*_i_=0.5±0.1 mm. Reactions were carried out at 28 °C under the optimised assay conditions and quenched after 3 min reaction time to measure the initial rate.

### Substrate specificity

To interrogate the substrate scope of Phe3H, a suite of non‐native substrates was screened under the optimised assay conditions. The electron‐rich substrate l‐Tyr was seen to be a very poor substrate, with only traces (<1 %) of hydroxylated product being detected by LC‐MS. Similarly, the bulkier substrate l‐Trp was also a poor substrate, giving <1 % conversion to hydroxylated product.

**Scheme 2:**
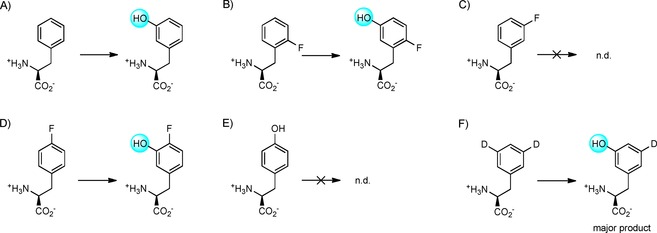
Regioselectivity of Phe3H‐catalysed hydroxylation of l‐Phe and l‐Phe derivatives. Reactions were carried out at 28 °C for 3 h with 5 μm purified Phe3H and 1 mm amino acid substrate in HEPES buffer containing catalase and superoxidase dismutase (SOD). Products were characterised by using LC‐MS and NMR spectroscopy.

Studies with dl‐2‐fluorophenylalanine (2F‐Phe), dl‐3‐fluorophenylalanine (3F‐Phe) and dl‐4‐fluorophenylalanine (4F‐Phe) revealed surprising results, with 2F‐Phe and 4F‐Phe being readily processed to the corresponding *meta* hydroxylated product. Akin to the observations with mammalian aromatic amino acid hydroxylases, Phe3H displayed very tight control of regiochemistry. As with l‐Trp and l‐Tyr, the reaction with 3F‐Phe only revealed products by LC‐MS analysis, with traces (<1 %) of hydroxylated fluorophenylalanine and *meta*‐Tyr being detected (Scheme 2). The latter compound is produced by dehalogenation, which is frequently observed for aromatic amino hydroxylases.^19^, ^20^, ^21^ In comparison to other hydroxylases, the low extent of defluorination seen for Phe3H is surprising. For example, rat TyrOHase converts 4F‐Phe to Tyr and 3F‐Tyr to DOPA.^6^ Similarly, the dehalogenation of 4F‐Phe to give tyrosine has also been reported for rat and sheep liver phenylalanine hydroxylases.^19^, ^20^ Further studies revealed 3F‐Phe to be a competitive inhibitor of Phe3H, with a *K*_i_ of 0.8 mm (Figure S8). It was postulated that 3F‐Phe might act as a product mimic due to the isosteric nature of the C−F and C−OH bonds. Furthermore, oxidation of the tetrahydropterin cofactor in the presence of only 3F‐Phe is the same as background oxidation, as is observed in the absence of any amino acid substrate (Figure S11), thus indicating that 3F‐Phe prevents binding of Phe to the active site and that it prevents formation of the active ferryl‐oxo species.

Although we observed l‐tyrosine to be a poor substrate, the almost isosteric 4F‐Phe proved to be a good substrate with an almost twofold lower *K*_M_ than that of l‐Phe (Table 1, entry 4 and Figure S7). The rate of the reaction was significantly slower for the fluorinated phenylalanines compared to nonfluorinated Phe; it was hypothesised that this is due to the weakly deactivating character of fluorine substituents in electrophilic aromatic substitution reactions. 2F‐Phe was also accepted as a substrate, albeit less efficiently than the 4F‐Phe analogue (Table 1, entry 5), with a single regioisomer product being generated in both cases (Scheme 2).

### Mechanism and NIH shift

Isotopic labelling has been used extensively to study the catalytic mechanisms of aromatic amino acid hydroxylases.^1^, ^4^, ^5^, ^22^, ^23^, ^24^ In this work, substrate deuterium labelling was employed to probe the mechanism of Phe3H.^1^, ^25^ [3,5‐D_2_]‐l‐Phe was synthesised in six steps from toluidine by enzymatic conversion of [3,5‐D_2_]phenylpyruvate to [3,5‐D_2_]‐l‐phenylalanine as the final step.^26^, ^27^ The final l‐phenylalanine consisted of 90 % D_2_, 10 % D_1_ and <1 % D_0_ l‐Phe as determined by integration of residual 5‐H signal in the ^1^H NMR spectrum and the relative intensities of isotope peaks in the mass spectrum. The steady‐state parameters for [3,5‐D_2_]‐l‐Phe were slightly slower than those of the nondeuterated Phe (Table 1, entry 6). To interrogate the mechanism further, [3,5‐D_2_]‐l‐Phe was incubated with Phe3H, and the products were isolated and analysed by ^1^H and ^2^H NMR spectroscopy (Figure S17). From these experiments, it was seen that the deuterium at the unreacted *meta*‐position C‐3 (7.18 ppm) was completely retained, as shown by the relative intensity of the residual C‐5−H signal. Aromatic amino acid hydroxylases have been demonstrated to operate by a mechanism that involves an NIH shift. In all previous mechanistic studies (typically with Trp and Phe hydroxylases) the deuterium or tritium label from the hydroxylation position was present in >60 % of the product.^1^, ^4^, ^22^, ^24^, ^25^ Unexpectedly, in this work with Phe3H, the second deuterium atom was lost. The almost complete loss of one deuterium from [3,5‐D_2_]‐l‐phenylalanine that we observe is therefore striking and suggests that a mechanism different from the conventional NIH shift operates for Phe3H.

### Mutational analysis

There are three possible mechanisms that could account for the loss of the deuterium label. A radical abstraction followed by oxygen rebound, akin to α‐ketoglutarate‐dependent dioxygenases, would result in loss of the *ipso*‐deuterium. Although a radical mechanism has recently been proposed for the Fe^II^dependent hydroxylase PqqB as a key step in the biosynthesis of the cofactor pyrroloquinoline quinone (PQQ), this enzyme does not use a pterin cofactor; rather, dioxygen is directly activated by the Fe^II^ centre.^28^ To date, there is no evidence to support a radical mechanism for iron‐ and pterin‐dependent hydroxylases.^1^, ^6^, ^29^ Alternatively, electrophilic aromatic substitution of the ferryl‐oxo species would yield the cationic intermediate. In the presence of a suitably positioned basic residue, the deuterium could be abstracted either in the *ipso* position or in the *ortho* position after 1,2‐hydride shift. If both the NIH shift and proton abstraction are stereospecific, this could account for our observed loss of the deuterium label.

The third option is direct deprotonation following electrophilic addition of the ferryl‐oxo species. For this to occur, the proton‐acceptor residue would have to be positioned close to the aromatic ring on the opposite side of the iron centre. C187 and T202 have previously been proposed as residues important in conferring regioselectivity for this enzyme.^10^ Walsh and co‐workers have previously reported the C187F mutant to be inactive and the T202G variant and the T202G/C187F double mutant to show a greater preference for hydroxylate at the *para* position, with this preference being most notable for the double mutant. It was proposed that both C187 and T202 act in concert to control the regiospecific hydroxylation of l‐Phe.^10^

Inspection of the alignment of Phe3H with the well‐characterised Phe4H and tyrosine 3‐hydroxylase homologues (Figure S22) revealed that C187 substitutes for the Phe residue found in Phe 4‐hydroxylases, Tyr 3‐hydroxylases and Trp 5‐hydroxylases. This Phe residue, together with a strictly conserved proline residue and a tryptophan residue (Phe in Trp hydroxylases), provides hydrophobic and π‐stacking interactions with the aromatic substrate side chain. It is noteworthy that all phenylalanine 3‐hydroxylases (from the napsamycin, sansanmycin, sanglifehrin pathways) contain Cys in the place of Phe. T202 of Phe3H is also specific to Phe 3‐hydroxylases and substitutes for the Gly residue that is present in Phe 4‐hydroxylases. T202 is located close to the putative substrate binding site in homology model studies (Figure S23).^10^

To probe the function of these residues, a panel of Phe3H C187 and T202 mutants was generated, and the kinetics werer measured with both l‐Phe and [D_2_]‐l‐Phe (Table 2). Intriguingly, C187A retained activity, albeit with lower *k*_cat_, and fourfold lower *K*_M_. C187S was also active, however the reaction rate was too slow to be determined by using Michaelis–Menten kinetics. Both mutants exclusively formed the *meta*‐Tyr isomer. The T202S mutant, which was designed to retain chemical reactivity whilst altering the steric environment, lost regiospecificity, with 20 % of the *para* product being observed, and catalysed the reaction at a much lower rate than the WT enzyme. Phe3H T202A displayed very slow hydroxylase activity, to the extent that it was not possible to measure the kinetic parameters. After prolonged reaction time, however, it was observed that both *para*‐ and *meta*‐Tyr had been produced, with *meta*‐tyrosine being produced at faster rate than *para*‐Tyr (Figure S24) giving an overall product distribution of 33 % *para*‐ and 67 % *meta*‐ (Table 2).

**Table 2:** Regioselectivity and kinetic parameters of hydroxylation of l‐Phe and [D_2_]Phe by Phe3H WT and Phe3H single‐point mutants. Time‐course experiments indicated C187S and T202A to be over 45 times slower than T202S; as such, accurate determination of *k*_cat_ and *K*_M_ for these mutants was not possible.

Enzyme	Regioselectivity	*k*_cat_ [min^−1^]		*K*_M_ [mm]	
		l‐Phe	[D_2_]Phe	l‐Phe	[D_2_]Phe
Phe3H	100 % *meta*	38±2	34±1	0.40±0.06	0.54±0.07
Phe3H C187A	100 % *meta*	17±0.3	13±0.4	0.1±0.01	0.13±0.03
Phe 3H C187S	100 % *meta*	n.d.	n.d.	n.d.	n.d.
Phe3H T202S	80 % *meta* 20 % *para*	26±5	13±4	0.5±0.2	0.4±0.3
Phe3H T202A	33 % *para* 67 % *meta*	n.d.	n.d.	n.d.	n.d.

**Scheme 3:**
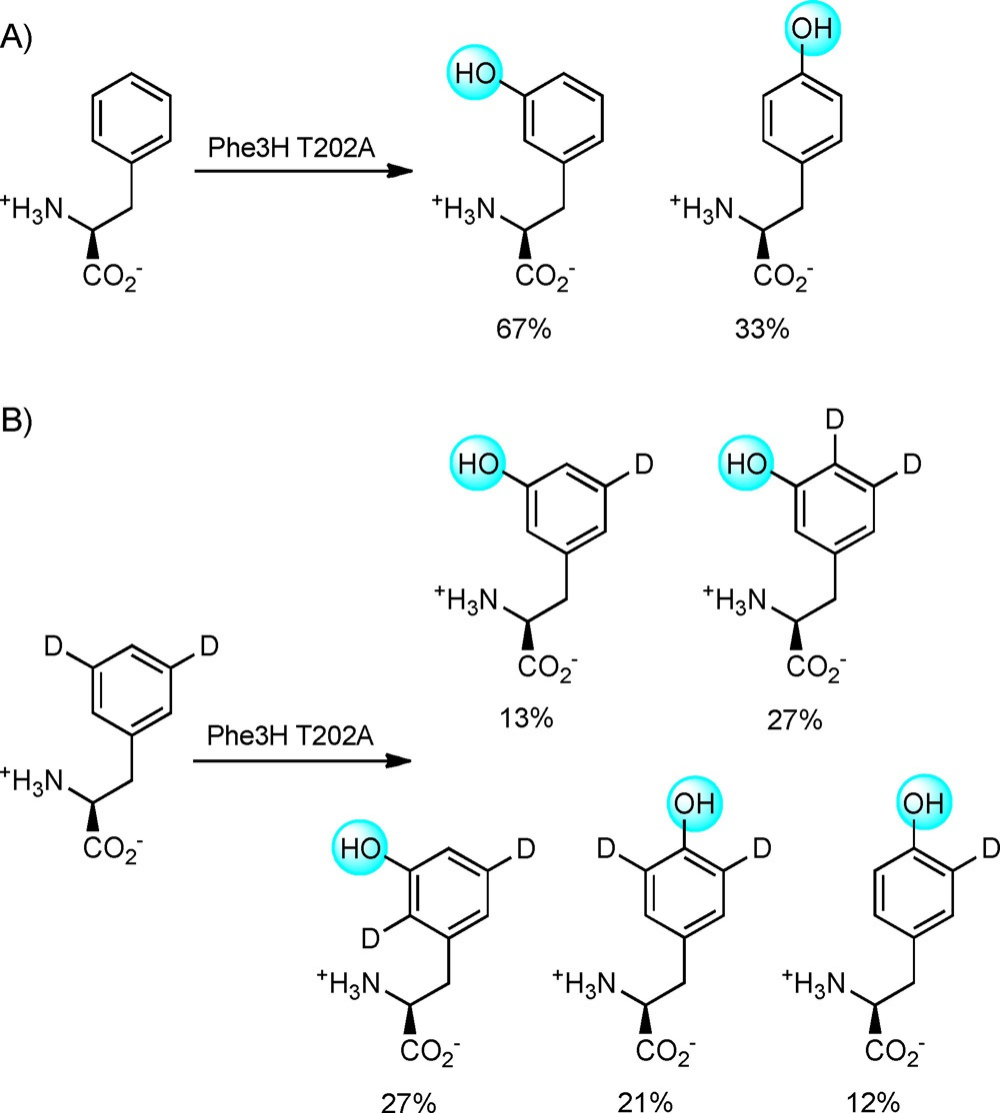
A) Regioselectivity of Phe3H T202A‐catalysed hydroxylation of l‐Phe. B) Regioselectivity of hydroxylation of [3,5‐D_2_]Phe by Phe3H T202A. Reactions were carried out at 28 °C for 3 h with 5 μm purified Phe3H and 1 mm amino acid substrate in HEPES buffer containing catalase and SOD. Products were characterised by using LC‐MS and NMR spectroscopy.

To gain a greater understanding of the process catalysed by this mutant, the two regioisomeric products of the Phe3H T202A‐catalysed reaction were isolated and studied by NMR spectroscopy. Intriguingly, for *meta*‐Tyr, 80 % of the product retained both deuterium labels, either at the 3, 4‐ or 2, 5‐positions, thus indicating that, for this mutant, the NIH shift mechanism must operate like that observed for Phe4Hs (Scheme 3). In the case of *para*‐Tyr, 63 % of the product retained both deuterium labels. This much higher level of retention than seen for the WT enzyme is again more consistent with the NIH‐shift mechanism that operates in Phe4Hs. It is noteworthy that, for the other mutant (T202S), the *meta*‐Tyr product obtained had lost the second deuterium label.

**Scheme 4:**
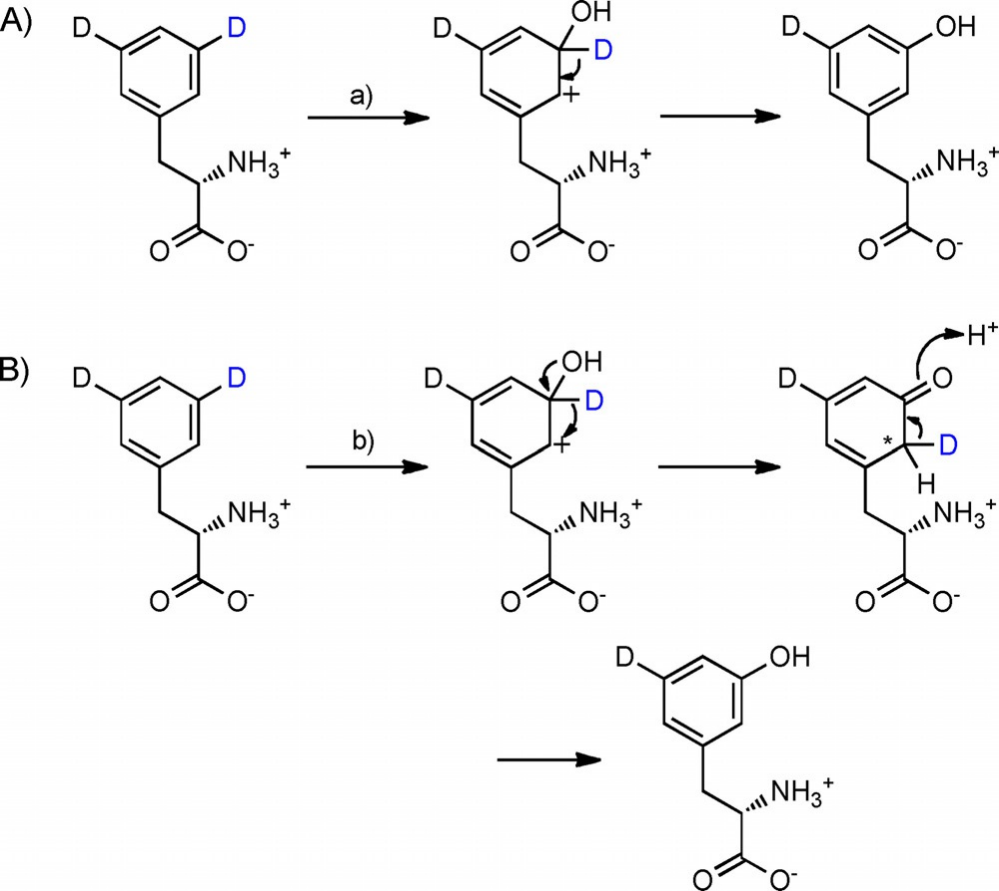
Two putative mechanisms of deprotonation following *meta*‐hydroxylation of Phe by WT Phe3H. A) Direct deprotonation or B) stereospecific NIH shift followed by stereospecific deprotonation to restore aromaticity would both account for the observed loss of one of the deuterium labels.

These data provide strong evidence that T202 acts as a switch between hydride shift (NIH mechanism) and direct deprotonation or stereospecific NIH shift and deprotonation. These two possible reactions of the cationic intermediate are shown in Scheme 4. Evidence from the kinetic and labelling studies in combination with the reduced efficiency of T202 mutants indicate that Thr‐OH coordination in combination with energetically favourable re‐aromatisation through deprotonation mediates this important distinction in mechanism compared to homologues such as the Phe4Hs bearing Gly rather than Thr. It is clear that T202 also plays an important role in directing the regiochemical outcome.

## Conclusion

To conclude, Phe3H from *S. coeruleorubidus* has been demonstrated to be an efficient enzyme for the production of *meta*‐tyrosine and to regiospecifically hydroxylate two fluorinated substrate analogues. Contrary to the established mechanism for phenylalanine *para*‐hydroxylase, deuterium labelling experiments showed that Phe3H from *S. coeruleorubidus* does not necessarily operate through an NIH shift mechanism. According to our isotopic labelling studies, the loss of the ferryl‐oxo species might occur without C−H bond migration or otherwise involves stereospecific NIH shift and deprotonation. Furthermore, investigation of the enzyme kinetics of T202A with l‐Phe and [D_2_]‐l‐Phe as the substrate revealed a significant isotope effect, as well as a partial change in regioselectivity, thus indicating that this residue plays an important role in the catalytic mechanism of this enzyme.

This insight into the catalytic mechanism of this unique set of enzymes could help to inform the design of biologically inspired catalysts for C−H bond functionalisation or rational engineering of hydroxylases to widen their substrate scope and synthetic utility.

## Conflict of interest

*The authors declare no conflict of interest*.

## Supporting information

As a service to our authors and readers, this journal provides supporting information supplied by the authors. Such materials are peer reviewed and may be re‐organized for online delivery, but are not copy‐edited or typeset. Technical support issues arising from supporting information (other than missing files) should be addressed to the authors.

Supplementary
