# Supplementary material for: Phenylalanine meta‐Hydroxylase: A Single Residue Mediates Mechanistic Control of Aromatic Amino Acid Hydroxylation
Source: Chembiochem. 2019 Jul 18;21(3):417–22. doi: 10.1002/cbic.201900320 (PMC7027792; doi:10.1002/cbic.201900320)
Supplement: Supplementary file 1 — Supplementary [file CBIC-21-417-s001.pdf]

CHEMBIOCHEM

Supporting Information

Phenylalanine *meta*-Hydroxylase: A Single Residue Mediates Mechanistic Control of Aromatic Amino Acid Hydroxylation

Sabine Grüschow,[a] Joanna C. Sadler,[a] Peter J. Sharratt,[b] and Rebecca J. M. Goss*[a]

cbic_201900320_sm_miscellaneous_information.pdf

Contents

1 Supplementary Figures 1
1.1 Materials and bacterial strains 3
1.2 General DNA manipulations 3
1.3 Heterologous production and purification of PhhA and PhhA single point mutants 3
1.4 Site directed mutagenesis for production of PhhA single point mutants 4
1.5 Enzyme assays 4
1.6 Kinetic analyses and inhibitor studies[1] 5
1.7 pH dependence 6
1.8 Non-enzymatic oxidation of pterin co-factor 6
1.9 Amino acid stereochemistry 6
1.10 LC-HRMS and HRMS analysis 6
1.11 NMR analysis 7
1.12 Synthesis 7
1.12.1 [2,6-d_2]-*para*-Toluidine 7
1.12.2 [3,5-d_2]-Toluene 7
1.12.3 [3,5-d_2]-Benzaldehyde 8
1.12.4 [3,5-d_2]-Phenylpyruvic acid 8
1.12.5 [3,5-d_2]-L-Phenylalanine 8
1.13 Enzyme reactions 9
1.13.1 *meta*-L-Tyrosine 9
1.13.2 [5-d_1]-*meta*-L-Tyrosine 9
1.13.3 2-Fluoro-5-hydroxy-L-phenylalanine 9
1.13.4 4-Fluoro-3-hydroxy-L-phenylalanine 10
2 Supplementary Figures 11
3 References 32

1 Supplementary Figures

Figure S1. SDS-PAGE analysis of His_6-Phe3H isolated from LB (A) and from TB (B) 12
Figure S2. Activity of Phe3H across pH 6.2-8.2 12

Figure S3. Inactivation of Phe3H by excess iron and pterin cofactor……….………………….………..13
Figure S4. Absorbance spectra showing non-enzymatic oxidation of $DMPH_4$……………………………14
Figure S5. Time course of Phe3H catalysed mTyr formation……………………………………………...15
Figure S6. Phe3H with Ni^{2+}, Zn^{2+} and Cu^{2+} inhibition curves……………………………………………...16
Figure S7. Steady-state (Michealis-Menten) kinetics of Phe3H catalysed reactions……………….………17
Figure S8. (a) Lineweaver-Burke plot of competitive inhibition of Phe3H by DL-3F-Phe. (b) Secondary plot of competitive inhibition of Phe3H by DL-3F-Phe…………………….……………………….……...18
Figure S9. UPLC analysis of the substrate scope of Phe3H…………….……………………………….19
Figure S10. LC-MS analysis of the substrate scope of Phe3H……………….……………………….20-21
Figure S11. Absorbance spectroscopy of oxidation of $DMPH_4$ in the absence and presence of substrate or inhibitor…………………………………………………………………….………………………..21
Figure S12. ^{19}F NMR (1H decoupled) of l-2-fluoro-5-hydroxyphenylalanine (A) and l-4-fluoro-3-hydroxyphenylalanine (B)……………….……………………………………………………….…….22
Figure S13. ^{1}H NMR (aromatic region) of l-2-fluoro-5-hydroxyphenylalanine in CD_3OD (A) without ^{19}F decoupling and (B) ^{19}F decoupled……………………………………………..………………23
Figure S14. ^{1}H NMR (A) and NOESY (B) of l-4-fluoro-3-hydroxyphenylalanine in CD_3OD…………….…24
Figure S15. ^{1}H NMR of L-*meta*-tyrosine obtained from Phe3H reaction with L-Phe as substrate…………...25
Figure S16. ^{1}H NMR of [d_1]-L-*meta*-tyrosine obtained from Phe3H reaction with L-[3,5-d_2]-Phe as substrate……………………………………………………………………………….………….25
Figure S17. Comparison of ^{1}H and ^{2}H NMR spectra in D_2O and H_2O/D_2O, respectively, of L-*meta*-tyrosine obtained from Phe3H reaction with L-[3,5-d_2]-Phe as substrate………………………..………………..26
Figure S18. HRMS analysis of L-*meta*-tyrosine obtained from Phe3H catalysed reaction with L-Phe as substrate……………………………………………………………………….…………………….27
Figure S19. HRMS analysis (ESI, -ve) of [d_1]-L-*meta*-tyrosine obtained from Phe3H reaction with L-[3,5-d_2]-Phe as substrate……………………………………………………………..…………………28
Figure S20. Determination of 4-fluoro-3-hydroxyphenylalanine stereochemistry using L-Marfey's reagent (A) and D-Marfey's reagent (B)…………………………………………………………….………29
Figure S21. Verification of [3,5-d_2]-phenylalanine stereochemistry using Marfey's reagent………………...30
Figure S22. Multiple sequence alignment of Phe4H from *Chromobacterium violaceum* with phenylalanine 3-hydroxylases from the pacidamycin, napsamycin, sansanmycin and sanglifehrin pathways, a hypothetical protein from *Streptomyces filamentosus NRRL 15998,* rat and human Phe4H and tyrosine *meta*-hydroxylase……………………………………………………………………………...…………..31
Figure S23. Model of Phe3H with Trp and BH_4 in the active site showing the position of putative base residues C187 and C202………………………………...………………………….………………32
Figure S24. Time course of mTyr and pTyr formation by Phe3H T202A……………………………...32
Figure S25. Comparison of rate of velocity of Phe3H catalysed mTyr formation with L-Phe and DL-Phe……..33
Table S1. Conversion of L-Phe to Tyr or mTyr in 3 hours reaction time at 28 °C……………….………33

Materials and Methods

1.1 Materials and bacterial strains

Microbiological media, buffer components, reagents, and resins were purchased from BD Biosciences (Oxford, UK), ForMedium™ (Hunstanton, UK), Melford (Chelsworth, UK), Sigma-Aldrich (Haverhill, UK), or Alfa Aesar (Hyesham, UK) and used without further purification. Formate dehydrogenase from *Candida boidinii* and phenylpyruvate dehydrogenase from *Sporosarcina* sp. were purchased from Sigma-Aldrich (Haverhill, UK). *Streptomyces coeruleorubidus* AB1183F-64 was obtained from the Agricultural Research Service Culture Collection, National Center for Agricultural Utilization Research (NRRL, Peoria, USA). *Streptomyces lividans* TK24 and *Streptomyces coelicolor* M1154 was provided by Prof. Mervyn J. Bibb (John Innes Centre, Colney, Norwich, UK). *Escherichia coli* DH10B-T1 (Invitrogen, Paisley, UK) was used for routine cloning, *E. coli* BL21(DE3) (Novagen, Merck Biosciences, Nottingham, UK) was used for protein expression.

1.2 General DNA manipulations

Restriction enzymes, ligase and *Pfu* DNA polymerase were obtained from Roche Diagnostics Ltd. (Burgess Hill, UK), Fermentas (St. Leon-Rot, D) or Promega (Southampton, UK) and used according to the manufacturer's instructions. Routine DNA sequencing was carried out by GATC Biotech Ltd. (London, UK).

1.3 Heterologous production and purification of PhhA and PhhA single point mutants

The *phhA* gene (encoding Phe3H) was PCR-amplified from *Streptomyces coeruleorubidus* AB1183F64 using primers 5'-ccatatgcaagggccgcacgccca and 5'-caagcttggctcagtgaggtgtcaccgagc and introduced into pJET1.2 (Fermentas, St. Leon-Rot, D). The gene of interest was excised with *Nde*I and *Hin*dIII and ligated into pET-28b(+) (Merck Biosciences, Nottingham, UK) to allow for expression of PhhA as a N-terminal His-tagged fusion protein. The construct was verified by DNA sequencing. *E. coli* BL21(DE3) was used as expression host in Lysogeny Broth (LB) containing 50 µg mL^{-1} kanamycin. Protein production was induced with 80 µM IPTG and carried out at 16 °C for 24 h. Harvested cells were resuspended in lysis buffer (0.5 M NaCl, 50 mM Tris-HCl, 10 mM imidazole, pH 8.0) supplemented with 100 µM ammonium iron(II) sulphate and 2 mM DTT. After cell lysis by sonication and removal of cell debris the soluble fraction was loaded onto Ni-NTA resin (Qiagen). The resin was washed with lysis buffer and eluted with lysis buffer containing 0.4 M imidazole. Protein containing fractions were pooled and DTT was added to a final concentration of 1 mM. PhhA was further purified on a HiLoad 16/60 Superdex 200 column (GE Healthcare, Little Chalfont, UK) in 20 mM HEPES, 200 mM NaCl, pH 7.5. His_6-PhhA elutes as a monomer. PhhA-containing fractions were combined, TCEP and glycerol were added to 0.25 mM and 10% final concentration, respectively. The protein solution was flash-frozen and stored at -80 °C. Approximately 3 mg His_6-PhhA were typically obtained from 1 L of culture.

The purity was assessed by SDS-PAGE analysis (Figure S1). Protein concentrations were determined by the Bradford method using BSA as the standard. To determine metal stoichiometry, protein concentrations were determined by amino acid analysis (PNAC facility, Department of Biochemistry, University of Cambridge,

UK); metal analysis was performed on a Varian Vista-PRO ICP-OES at the School of Environmental Sciences, University of East Anglia, UK or by ICP-MS at the University of Edinburgh.

1.4 Site directed mutagenesis for production of PhhA single point mutants

Site-directed mutagenesis was accomplished by a modified QuickChange method using primers bearing desired mutations. The PCR contained 1X HF buffer (ThermoScientific), 2.5 mM dNTPs, 5% v/v DMSO, 1-10 ng template DNA, 250 nM of each primer, 0.5 µL Phusion HiFi DNA polymerase (New England Biolabs) and MilliQ water to a final volume of 50 µL. PCR was carried out using the following parameters: 98 °C for 30 seconds, then 20 cycles of 98 °C for 15 seconds, 60 °C for 45 seconds and 72 °C for 6 minutes, then 10 minutes at 72 °C. PCR reactions were then treated with Dpn1 and 5 µL of the resultant mixture was used to transform 100 µL DH10B-T1 competent cells. Plasmid DNA was isolated by MiniPrep (Qiagen) and the gene of interest verified by Sanger sequencing using T7 forward and T7 reverse primers. Protein expression was carried out using *E. coli* BL21 (DE3) as the host strain as described for above.

1.5 Enzyme assays

Optimisation of Phe3H assay conditions

Prior to measuring the Michaelis-Menten kinetics of Phe3H, we sought to optimise the assay conditions and identify potential inhibitors of the enzyme. Phe3H was expressed heterologously in *E. coli* and purified by Ni-affinity and size exclusion chromatography (Figure S1). The resultant enzyme was screened for activity in MES, HEPES and Tris buffers across their pH ranges. In contrast to previously reported data,[1] only baseline levels of activity were detected below pH 7, with the optimal tested conditions being HEPES buffer at pH 8. Swapping to Tris buffer at pH 8 resulted in a 2.5-fold decrease in enzyme activity (Figure S2). This result has also been reported for rat and bovine PhhA, in which it was proposed that Tris may coordinate directly to the active ferric iron centre, thereby decreasing the rate of Fe(III) reduction.[2,3]

The influence of additives such as ascorbate, excess ferrous iron, catalase and superoxide dismutase (SOD) was also investigated. SOD and catalase were found to rescue the activity of Phe3H in the presence of excess Fe(II) and protect the tetrahydropterin cofactor from non-enzymatic oxidation, further improving the efficiency of this reaction (Figures S3-S5). Ascorbate and dithiothreitol (DTT) were also found to slow down the non-enzymatic oxidation of the pterin cofactor. As DTT can scavenge Fe(II) from the active site of the enzyme, however, it was omitted from the enzymatic assays.[3–5]

Substrate scope experiments

The assay mixture contained Phe3H (5 µM), amino acid (1 mM), catalase (10 U μL^{-1}), and SOD (0.1 U μL^{-1}) in 40 mM HEPES, pH 8.0. Reactions (in duplicate) were started by addition of $6MPH_4$ (1 mM) and incubated at 28 °C for 3 h. Enzyme was removed by ultrafiltration using VWR centrifugal filters (modified PES membrane, MWCO 10 kDa). The flow-through was analysed by UPLC as described below and by LC-MS.

Phe3H reaction mixtures for kinetic analysis were carried out at least in triplicate and contained 0.5 µM Phe3H, 0.5 mM $6MPH_4$ or $DMPH_4$, 1 U μL^{-1} catalase, 0.01 U μL^{-1} superoxide dismutase and varying amounts of L-Phe (or [3,5-d_2]-L-Phe, 2F-DL-Phe or 4F- DL-Phe) in 40 mM HEPES, pH 8.0. Alternatively, the L-Phe concentration

was kept constant (5 mM) and the concentration of $6MPH_4$ or $DMPH_4$ was varied. Reactions were started by the addition of $6MPH_4$ or $DMPH_4$ after preincubation at 28 °C for 6 – 8 min. Reactions were carried out at 28 °C and were quenched after 3 min with an equal volume of 1% trifluoroacetic acid. IC_{50}s for metal and DL-3F-Phe inhibition were determined using the same conditions as for kinetic analysis using $6MPH_4$ as cofactor with the addition of the indicated metal ions (0.001 µM – 1 mM) at a fixed L-Phe concentration (5 mM), or by varying both L-Phe (0.2 – 5 mM) and DL-3F-Phe (1.2 – 10 mM) concentrations, respectively. Samples were subjected to HPLC analysis (Waters Acquity® H-Class UPLC equipped with photodiode and fluorescence detectors). Compounds were separated on a Waters Acquity UPLC® BEH C18 column (1. 7 µm, 2.1 x 50 mm) with 50 mM triethylamine : 60 mM trifluoroacetic acid, pH 2.0 as buffer A and methanol as solvent B (0–0.4 min 3% B, 0.4–2.2 min 3-18% B, 2.2–2.4 min 18-60% B, 2.4-3.2 min 60% B, 3.2–3.4 min 60-3% B, 3.4–4.6 min 3% B; flow rate 0.5 mL min^{-1}). Products were detected using fluorescence measurements (λ_{ex} 275 nm, λ_{em} 310 nm) and quantified by comparison to standard curves (2.5 – 200 µM). For mTyr commercially available *meta*-DL-Tyr was used as standard, whereas for quantitation of fluorinated *m*Tyr, compounds were purified from enzyme reactions and quantified by qNMR using maleic acid as standard.

1.6 Kinetic analyses and inhibitor studies[1]

Non-linear regression was performed using Prism software. For steady-state kinetics the data points (4 – 6 replicates) were fitted to

$$v_0 = \frac{V_{\max} \cdot [S]}{K_M + [S]} \qquad \text{Eq. 1}$$

where v_0 is the initial rate (µM product min^{-1}), V_{max} is the maximal reaction velocity, K_M is the Michaelis-Menten constant. The rate constant k_{cat} was calculated as V_{max} / [Phe3H].

The associated error in k_{cat} and K_M values were calculated using the following equation[6,7]:

$$\Delta\left(\frac{k_{cat}}{K_M}\right) = \left(\frac{k_{cat}}{K_M}\right) \cdot \sqrt{\left(\frac{\Delta k_{cat}}{k_{cat}}\right)^2 + \left(\frac{\Delta K_M}{K_M}\right)^2} \qquad \text{Eq.2}$$

For steady state kinetics with substrate inhibition, data were fitted to

$$v_0 \frac{V_{max} * [S]}{K_M + ([S] * \left(1 + \frac{[S]}{K_i}\right))} \qquad \text{Eq. 3}$$

where K_i is the inhibitor binding constant for reversible inhibitors.

For inhibition by DL-3F-Phe, the measured rates were presented as Lineweaver-Burk plots for each inhibitor concentration. Slopes were determined by linear regression and plotted against the inhibitor concentration to give the secondary plot. Linear regression was used to determine the X axis intercept $x_{y=0}$, with the $IC_{50} = - x_{y=0}$.

1.7 pH dependence

Potassium phosphate buffers were prepared at 100 mM for pH 5.8, 6.3, 6.6, 7.0, 7.5, 7.9, 8.5, and were used at 50 mM final concentration. Reactions contained 0.5 μM Phe3H, 5 mM L-Phe, and were started by the addition of 0.1 mM $DMPH_4$ after preincubation at 28 °C for 5-7 minutes. Reactions were conducted in triplicate with 100 μL reaction volume in 96-well plates (Nunc). The formation of mTyr was monitored by measuring the change in fluorescence at λ_{em} 305 nm (λ_{ex} 270 nm) using a Molecular Devices SpectraMax® M5 instrument. In an independent experiment, MES, HEPES and Tris buffers were prepared to 60 mM ionic strength (calculated using the Buffer Calculator tool on www.biomol.net) to give pH 6.2 and 6.8 (106 mM or 72 mM MES, respectively), pH 6.8, 7.4, and 8.0 (385 mM, 142 mM, and 81 mM HEPES, respectively), pH 8.0, 8.4, and 8.8 (100 mM, 160 mM, and 310 mM Tris, respectively). The final ionic strength in the reactions was 30 mM for each buffer. Enzyme reactions were conducted in triplicate at 28 °C and contained 0.5 μM Phe3H, 1 mM ascorbate, 1 mM L-Phe. The reactions were preincubated for 5-10 minutes, started by addition of 0.5 mM $6MPH_4$ and stopped after 5 min by addition of an equal volume of 1% aq. TFA. Reactions were analysed by UPLC (Waters Acquity UPLC® BEH C18 column) as described in the above.

1.8 Non-enzymatic oxidation of pterin co-factor

Reactions were conducted in duplicate at 28 °C in Nunc 96-well plates with a 200 μL reaction volume. The oxidation of $DMPH_4$ to $DMPH_2$ was monitored by recording the increase in absorbance at 340 nm using a FLUOstar Omega plate reader (BMG Labtech). All reactions contained 50 mM HEPES, pH 7.5 and were started by the addition of 0.2 mM $DMPH_4$. The following additives were tested alone or in combination: 1 mM DTT, 1 mM ascorbate, 10 μM ferrous ammonium sulfate/100 μM DTT or 5 μM ferrous ammonium sulfate/50 μM DTT, 0.05 U μL^{-1} superoxide dismutase, 5 U μL^{-1} catalase. $DMPH_2$ was generated from spontaneous oxidation of $DMPH_4$ in HEPES buffer, its identity was verified by UV spectroscopy. Results are shown in Figure S4.

1.9 Amino acid stereochemistry

The absolute stereochemistry was determined by derivatisation with Marfey's reagent according to published procedures.[2–4] Both L-fluoro-2,4-dinitrophenyl-5-L-alanine amide and L-fluoro-2,4-dinitrophenyl-5-D-alanine amide were reacted with 4-fluoro-3-hydroxyphenylalanine to the doubly derivatised product. For d_2-phenylalanine only the L- reagent was used.

1.10 LC-HRMS and HRMS analysis

A Velos Pro hybrid Orbitrap-Linear Ion Trap instrument equipped with a Dionex 3000 liquid chromatography system (Thermo Scientific) was used for LC-HRMS analysis. Enzyme reaction mixtures were separated on a Waters XBridge™ C18 column (3.5 μm, 2.1 × 150 mm) at 40 °C using 0.1% formic acid in water as solvent A and methanol as solvent B with the following gradient: 0 – 0.5 min 3% B, 0.5 – 8.5 min 3-18% B, 8.5-9.0 min 18 – 60% B, 9.0 – 11.0 min 60% B, 11.0 – 11.5 min 60 – 3% B, 11.5 – 14.5 min 3% B at a flow rate of 0.35 mL min^{-1}. Mass analysis was performed using the Orbitrap at 30,000 resolution in positive ion mode; the electrospray source was set to 3.5 kV source voltage, 350 °C capillary temperature, 250 °C source heater temperature.

HRMS analysis of pure compounds was provided by the EPSRC National Mass Spectrometry Facility (Swansea, UK) or performed in-house on the above instrument. The mass error (ppm) and the relative peak intensity (%) are indicated.

1.11 NMR analysis

Deuterated solvents were obtained from Apollo (Manchester, UK), Cambridge Isotope Laboratories (Andover, USA), or Sigma-Aldrich (Haverhill, UK). Spectra were recorded on Bruker 300 MHz, 400 MHz, or 500 MHz instruments and referenced to residual solvent signal (chloroform 7.26 ppm, water 4.79 ppm, dimethylsulfoxide 2.50 ppm). Chemical shifts are reported in ppm; abbreviations for multiplicities are as follows: s singlet, d doublet, t triplet, dd doublet of doublet, m multiplet; app apparent, os overlapping signal.

1.12 Synthesis

1.12.1 [2,6-d_2]-*para*-Toluidine

The title compound was prepared as described by Charlton *et al.* and Best and Wilson.[5,6] Toluidine (25 g, 0.23 mol) converted to the hydrochloride salt by heating it to reflux in ethanol (62 mL) and 37% HCl (75 mL) for 15 minutes, cooling the solution and collecting the precipitate by filtration. Toluidine hydrochloride was washed with cold water, then acetone and air dried for 1 hour. Toluidine hydrochloride was heated to reflux in 50 mL D_2O under nitrogen atmosphere overnight. The reaction was concentrated under reduced pressure and the procedure repeated twice. The final title compound was briefly washed with acetone after removal of D_2O and dried to give a slightly brown solid in 57% yield (0.13 mol). Deuterium exchange was 86% according to ^{1}H NMR. The regioselectivity of the deuteration was verified by observing an nOe from toluidine-CH_3 to 2-H & 6-H). ^{1}H NMR (300 MHz, CD_3OD) δ 7.24 (s, 2H, 3-*H* & 5-*H*), 7.20 (d, *J* = 8.8 Hz, residual 2-*H* & 6-*H*), 2.28 (s, 3H). ^{13}C NMR (126 MHz, CD_3OD) δ 139.2, 130.3, 127.7, 122.5 (residual 2-*C*H & 6-*C*H), 122.3 (t, *J* = 24.7 Hz), 19.6. HRMS (APCI, +ve) calculated for $C_7H_8D_2N$ (M+H$^+$) 110. 0933, found 110.0930 (-3.0 ppm).

1.12.2 [3,5-d_2]-Toluene

The method described by Fries and Imbert was adapted as follows.[7] To an aqueous HCl solution (1 N, 35 mL) was added [2,6-d_2]-*para*-toluidine (9.3 g, 62.6 mmol, 1 eq.), and the mixture was heated to 70 °C for 10 minutes. The solution was then cooled to 0 °C and sodium nitrite (5.15 g, 74.6 mmol, 1.2 eq., in 25 mL distilled water) was added dropwise over 90 minutes whilst keeping the temperature of the reaction mixture below 4 °C. During the course of the addition, the mixture changed first to a pink/red solution before turning into a yellow-brown suspension. Reductive removal of the diazo group was achieved by slowly adding of hypophosphorous acid (41 g of a 50 % solution in water; 0.31 mol, 5 eq. H_2POH) over a period of 30 min, taking care that the reaction temperature remained below 4 °C. The suspension was stirred on ice for another 60 minutes, then left to stand at 4 °C overnight before extracting three times with dichloromethane. The combined organic layers were dried ($MgSO_4$) and the combined organic fractions concentrated under reduced pressure. Distillation afforded the title compound as a clear liquid (3.16 g, 30 mmol, 48% yield) containing approximately 10% residual dichloromethane. ^{1}H NMR (400 MHz, $CDCl_3$) δ 7.23 (s, 2H), 7.22 (s, 1H), 2.41 (s, 3H). ^{13}C NMR (126 MHz,

$CDCl_3$) δ 139.7, 129.1 (residual non-deuterated toluene), 129.0, 128.3 (residual non-deuterated toluene), 128.0 (t, J = 24.4 Hz), 125.2 (residual non-deuterated toluene), 125.1, 21.5.

1.12.3 [3,5-*d₂*]-Benzaldehyde

The procedure was adapted from Zhao.[8] A solution of cerium(IV) ammonium nitrate (35 g, 64 mmol, 2 eq.) in 3.5 M nitric acid (130 mL) was added to [3,5-d_2]-toluene (3.0 g, 32 mmol, 1 eq.) and the orange solution was heated to 80 °C for 2 hours. The yellow solution was cooled to room temperature, then diethylether (100 mL) was added and the organic phase was washed with distilled water until the aqueous layer was pH 7. The organic layer was dried ($MgSO_4$) and the solvent carefully removed *in vacuo*. This procedure was repeated and the crude benzaldehyde extracts were combined. Distillation afforded the title compound as a yellow liquid that contained 0.5 eq. starting material. The corrected yield was 12% (1.31 g, 8 mmol). ^{1}H NMR (300 MHz, $CDCl_3$) δ 10.06 (s, 1H), 7.92 (s, 2H), 7.67 (s, 1H). ^{13}C NMR (126 MHz, $CDCl_3$) δ 192.5, 137.9, 134.5, 129.7, 128.6 (app. d, J = 24.7 Hz). HRMS (APCI, +ve) calculated for $C_7H_5D_2O$ (M+H$^+$) 109. 0617, found 109.0613 (-3.6 ppm).

1.12.4 [3,5-*d₂*]-Phenylpyruvic acid

The procedure was adapted from patent literature.[9,10] In two 1 mL reaction vials was assembled each: hydantoin (250 mg, 2.5 mmol, 1.4 eq.), [3,5-d_2]-benzaldehyde (260 mg containing 0.5 eq [3,5-d_2]-toluene, 1.8 mmol, 1 eq.), ethanolamine (15 µL, 0.25 mmol, 0.1 eq.), and 0.6 mL MQ-water. The vials were sealed, immersed in mineral oil and heated to 110 °C. The suspension initially dissolved upon heating of the mixture; after 5 hours at 110 °C, [3,5-d_2]-benzylidenehydantoin precipitated and the mixture was allowed to cool to room temperature. The suspension from both reaction vials was combined in a 50 mL round-bottom flask, the vials rinsed with MW-water (1 mL) and an aqueous NaOH solution (5 mL of a 5 N solution, 25 mmol NaOH, 7 eq.) was added. The reaction was heated to reflux under nitrogen atmosphere for 90 minutes The resulting solution was cooled to 20 °C and the pH carefully adjusted to 8.5 with concentrated aqueous HCl solution. NaCl (1.5 g) was added to aid precipitation and the mixture was left standing overnight. The solids were collected in a scintered funnel, washed with ice cold distilled water (3 mL) and methanol (3 mL). The filter cake was redissolved in distilled water and freeze-dried to give [3,5-d_2]-phenylpyruvate sodium salt as off-white solid (321 mg, 1.6 mmol, 45% yield over both steps). ^{1}H NMR (300 MHz, d_6-DMSO) δ 7.53 (s, enol form, 2'-*H*), 7.12 (s, 4'-*H*), 7.09 (s, keto form, 2'-*H*), 5.96 (s, enol form, 3-*H*), 3.79 (s, keto form, 3-H_2). ^{13}C NMR (101 MHz, d_6-DMSO, signals for keto- and enol-form) δ, 168.5, 167.5, 149.1, 137.8, 135.8, 130.1, 128.2, 126.3, 125.0, 99.6, 46.0. HRMS (ESI, -ve) calculated for $C_9H_5D_2O_3$ (M-H$^+$) 165.0528, found 165.0528 (-0.2 ppm).

1.12.5 [3,5-*d₂*]-L-Phenylalanine

The procedure from Raap *et al.* was used.[11] The reaction was carried out in ammonium formate buffer (1 M, 5.0 mL, pH adjusted to 8.6 with sodium hydroxide) containing [3,5-d_2]-phenylpyruvate sodium salt (200 mM final concentration), β-NAD$^+$ (1 mM), formate dehydrogenase (2 U mL^{-1}) and phenylpyruvate dehydrogenase (2 U mL^{-1}). After gentle shaking at 28 °C for 22 hours, the reaction mixture was loaded onto Dowex® 50WX8 resin (hydrogen form, 10 mL bed volume) and the resin was washed with distilled water (35 mL) before eluting with 1 M ammonium hydroxide (70 mL). Product-containing fractions were lyophilised to provide the title

compound as white solid (129 mg, 0.8 mmol, 79% yield). ^{1}H NMR (300 MHz, D_2O) δ 7.24 (s, 1H), 7.20 (s, 2H), 3.85 (dd, *J* = 7.8, 5.2 Hz, 1H), 3.16 (dd, *J* = 14.5, 5.2 Hz, 1H), 2.98 (dd, *J* = 14.5, 7.9 Hz, 1H). ^{13}C NMR (101 MHz, D_2O) 174.2, 135.1, 129.0 (t, *J* = 24.7 Hz), 128.5 (d, *J* = 25.0 Hz), 127.4, 56.1, 36.5. HRMS (ESI, +ve) calculated for $C_9H_{12}NO_2$ ($M+H^+$) 166.0863, found 166.0861 (-0.9 ppm, 1.3%); calculated for $C_9H_{11}DNO_2$ ($M+H^+$) 167.0925, found 167.0924 (-0.8 ppm, 22.8%); calculated for $C_9H_{10}D_2NO_2$ ($M+H^+$) 168. 0988, found 168.0983 (-3.0 ppm, 100.0%).

1.13 Enzyme reactions

For NMR analysis of reaction products, the Phe3H reaction was scaled up to 2 – 6 mL. The reaction mixture consisted of 9 μM Phe3H, 2 mM ascorbate, 0.05 U μL^{-1} superoxide dismutase, 5 U μL^{-1} catalase, 10 μM ferric ammonium sulphate / 100 μM DTT, 5 mM substrate in 50 mM Tris-HCl, pH 8.0 and 2 mM $DMPH_4$ for 2- and 4-fluoro-DL-phenylalanine as substrate. For unlabelled and isotopically labelled L-phenylalanine the reaction mixture consisted of 10 μM Phe3H, 1 mM ascorbate, 5 mU μL^{-1} superoxide dismutase, 5 U μL^{-1} catalase and 5 mM substrate in 50 mM HEPES, pH 8.0 and 1 mM $6MPH_4$. The reaction was incubated at 28 °C for 6 – 12 hours, then stopped by the addition of TFA to 1% final concentration. Products were isolated on a Luna 5 μm C18(2) column (Phenomenex, 100 Å, 21.2 × 250 mm) using a Gilson 322 HPLC system equipped with a UV/Vis-151 detector (compound detection at 275 nm) and fraction collector. Separation was achieved using a linear gradient of 5 – 90% methanol against 0.1% TFA in water over 38 min at 7 mL min^{-1} flow rate with a 5 minute delay before the start of the gradient. Solvent was removed by evaporation under reduced pressure, the residue was redissolved in 100 mM aqueous HCl and lyophilised to produce the title compounds.

1.13.1 *meta*-L-Tyrosine

^{1}H NMR (400 MHz, D_2O, acidic) δ 7.18 (t, *J* = 7.9 Hz, 1H), 6.73 – 6.77 (m, 2H), 6.70 (app t, *J* = 2.0 Hz, 1H), 4.01 (dd, *J* = 7.9, 5.3 Hz, 1H), 3.15 (dd, *J* = 14.5, 5.3 Hz, 1H), 2.99 (dd, *J* = 14.5, 8.0 Hz, 1H). ^{13}C NMR (126 MHz, D_2O) δ 173.5, 155.8, 136.7, 130.4, 121.4, 116.0, 114.6, 55.6, 36.0. HRMS (ESI, -ve) calculated for $C_9H_{10}NO_3$ $[M-H]^-$ 180.0666, found 180.0669 (1.4 ppm).

1.13.2 [5-d_1]-*meta*-L-Tyrosine

^{1}H NMR (400 MHz, D_2O, acidic) δ 7.18 (t, *J* = 7.9 Hz, 0.1H), 6.74 – 6.76 (m, 2H), 6.70 (app t, *J* = 1.7/2.5 Hz, 1H), 3.99 (dd, *J* = 7.9, 5.3 Hz, 1H), 3.15 (dd, *J* = 14.5, 5.3 Hz, 1H), 2.99 (dd, *J* = 14.6, 7.9 Hz, 1H). ^{2}H NMR (77 MHz, referenced to residual HDO at 4.70 ppm) δ 7.18 (1H, peak width at 10% 23 Hz), 6.71 (0.01H). ^{13}C NMR (126 MHz, D_2O) δ 172.8, 156.1, 136.5, 130.7 (from residual C5-H mTyr, C5-^{2}H triplet at s/n threshold), 121.7 (from residual C5-H mTyr), 121.6, 116.3, 115.0 (from residual C5-H mTyr), 114.9, 55.3, 36.0. HRMS (ESI, -ve) calculated for $C_9H_9DNO_3$ $[M-H]^-$ 181.0729, found 181.0734 (2.7 ppm).

1.13.3 2-Fluoro-5-hydroxy-L-phenylalanine

^{1}H NMR (500 MHz, MeOD, acidic) δ 7.00 (t, *J* = 9.0 Hz, 1H, H^3), 6.73 – 6.77 (m, 2H, $H^{4,6}$), 4.21 (dd, *J* = 7.8, 5.8 Hz, 1H), 3.31 – 3.35 (os), 3.11 (dd, *J* = 14.4, 8.0 Hz, 1H). ^{13}C NMR (500 MHz, HMBC, MeOD) δ, 169.5,

155.9, 154.6, 153.7, 121.8, 117.3, 115.2, 52.8, 29.9. ^{19}F NMR (500 MHz, MeOD, ^{1}H decoupled) δ -132.0 (s). HRMS (ESI, +ve) calculated for $C_9H_{11}FNO_3$ $[M+H]^+$ 200.0718, found 200.0713 (-2.5 ppm).

1.13.4 4-Fluoro-3-hydroxy-L-phenylalanine

^{1}H NMR (400 MHz, MeOD, acidic) δ 6.94 (dd, $^{3}J_{HF}$ 11.0 Hz, $^{3}J_{HH}$ 8.3 Hz, 1H, H^5), 6.78 (dd, $^{4}J_{HF}$ 8.3 Hz, $^{4}J_{HH}$ 2.1 Hz, 1H, H^2), 6.62 (ddd, $^{3}J_{HH}$ 8.3 Hz, $^{4}J_{HF}$ 4.1 Hz, $^{4}J_{HH}$ 2.3 Hz, 1H, H^6), 4.10 (dd, J = 7.7, 5.4 Hz, 1H), 3.12 (dd, J = 14.5, 5.3 Hz, 1H), 2.96 (dd, J = 14.6, 7.8 Hz, 1H). ^{13}C NMR (400 MHz, HMBC, MeOD) δ 170.7, 153.4, 151.7, 146.6, 131.7, 121.4, 119.7, 54.8, 36.6. ^{19}F NMR (400 MHz, MeOD, ^{1}H decoupled) δ -140.9 (s). HRMS (ESI, +ve) calculated for $C_9H_{11}FNO_3$ $[M+H]^+$ 200.0718, found 200.0715 (-1.1 ppm).

2 Supplementary Figures

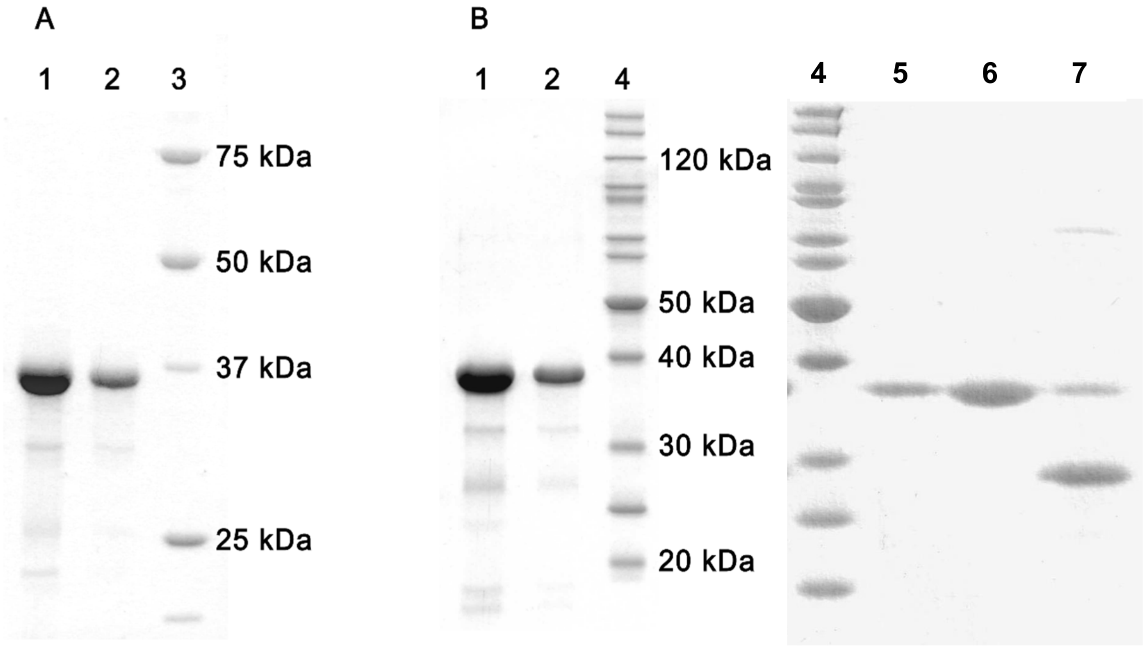

Figure S1. SDS-PAGE analysis of His$_6$-Phe3H isolated from LB (A) and from TB (B). Final His$_6$-Phe3H solution (lane 1), four-fold diluted protein solution (lane 2), Promega Broad Range Protein Molecular Weight Marker (lane 3), Fermentas PageRuler (lane 4). The resulting protein was further purified by gel filtration (C). Fractions containing pure Phe3H (lanes 5 and 6) were pooled and used in enzyme assays.

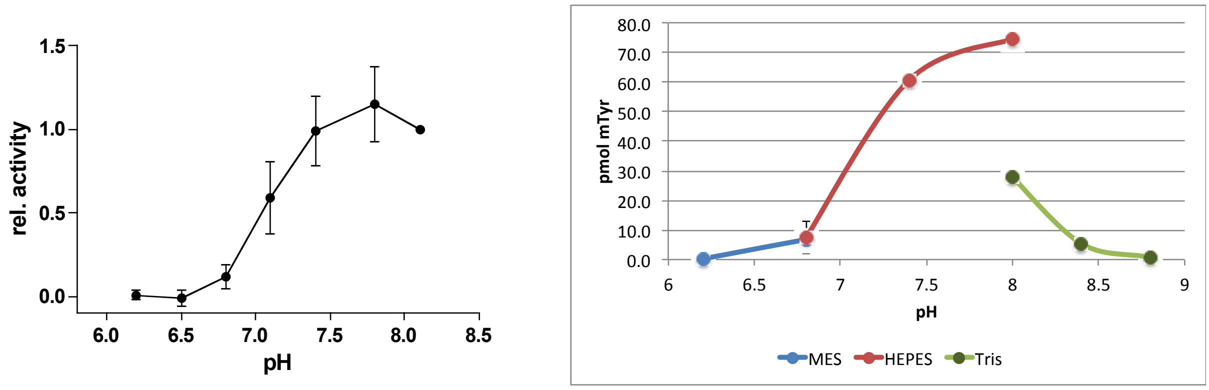

Figure S2. Activity of Phe3H across pH 6.2-8.2 in (A) phosphate buffers and (B) Mes, HEPES and Tris buffers as indicated.

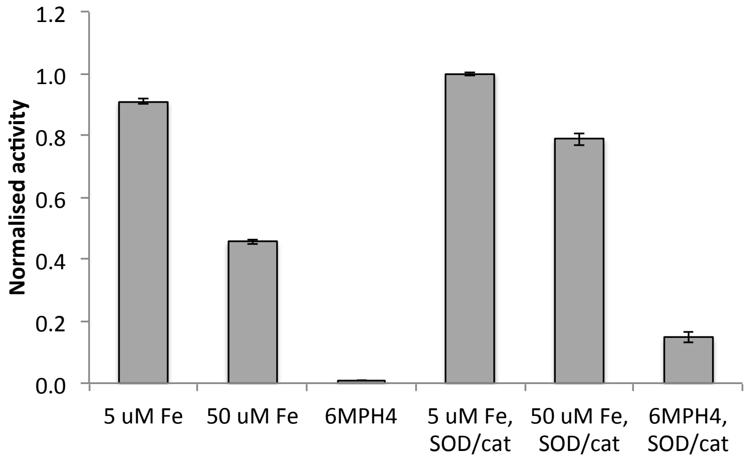

Figure S3. Inactivation of Phe3H by excess iron and pterin cofactor. Reaction conditions: Phe3H (0.5 μM) was incubated for 10 min at 28 °C with Fe^{2+} (5 μM ferrous ammonium sulfate / 50 μM DDT and 50 μM ferrous ammonium sulfate / 0.5 mM DDT) or $6MPH_4$ (0.5 mM) in the presence or absence of catalase and SOD (1 U μL^{-1} and 0.01 U μL^{-1}, respectively) in 40 mM HEPES, pH 8.0. The substrate (1 mM L-Phe), fresh catalase / SOD (1 U μL^{-1} / 0.01 U μL^{-1}) and $6MPH_4$ (0.5 mM) were added, the reaction incubated for a further 3 minutes before quenching with an equal volume of 1% TFA in water. Samples were analysed as described above. Results shown are from duplicate experiments and represent the normalised amount of product observed.

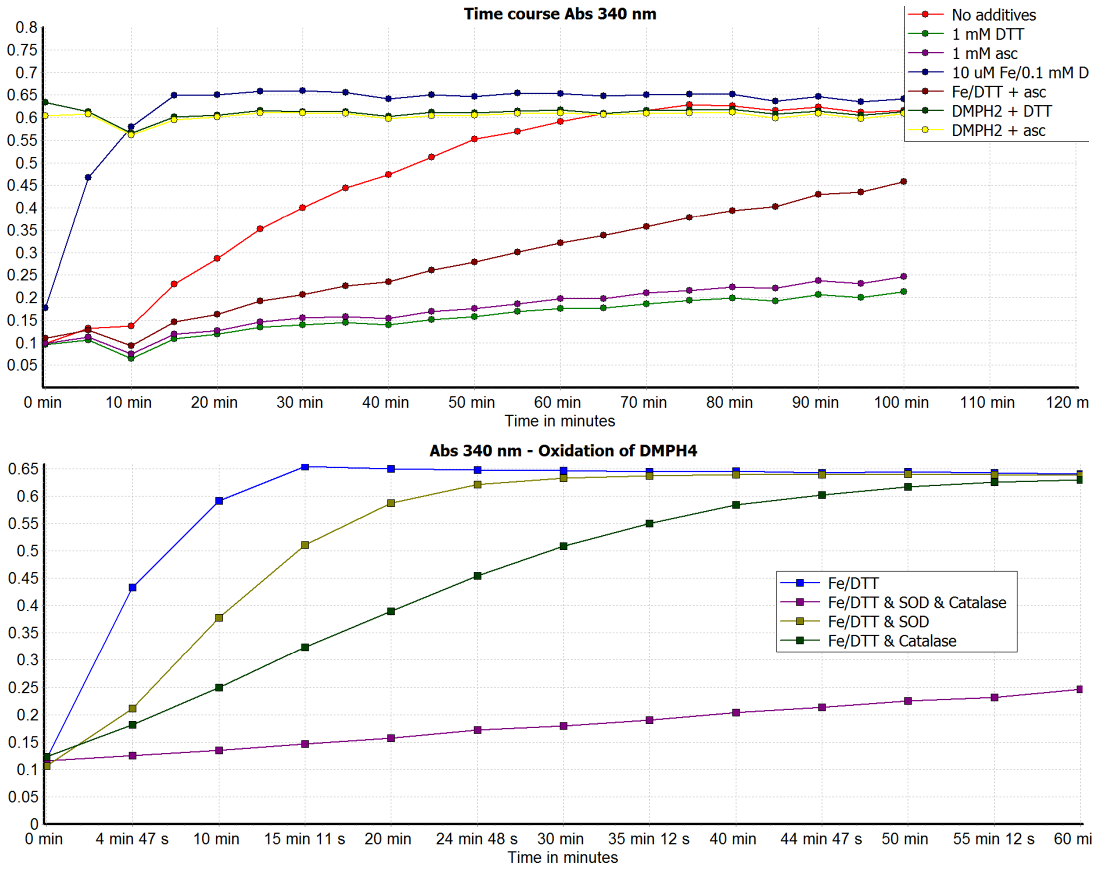

Figure S4. Absorbance spectra showing non-enzymatic oxidation of $DMPH_4$. The absorbance at 340 nm increases upon oxidation of $DMPH_4$ to $DMPH_2$.

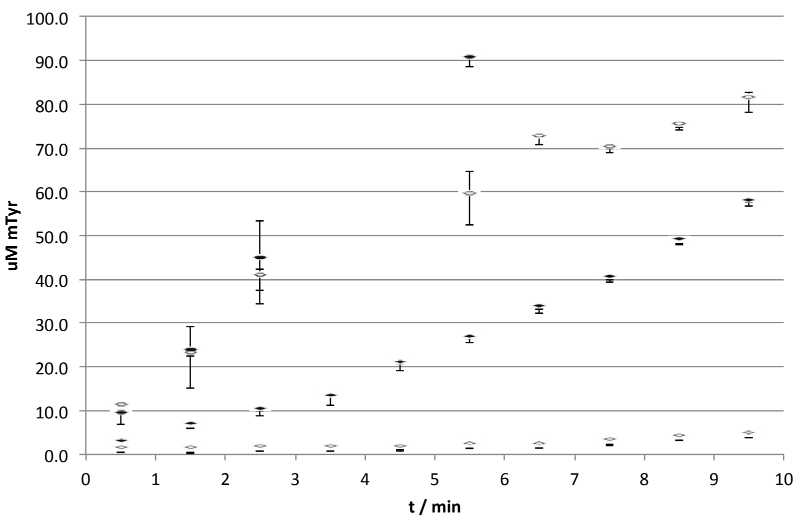

Figure S5. Time course of Phe3H catalysed mTyr formation. Reaction conditions: 0.5 μM Phe3H, 5 mM L-Phe, 0.5 mM 6MPH$_4$ in 40 mM HEPES, pH 8.0. The reactions were incubated at 28 °C and quenched at indicated time points with an equal volume 1% trifluoroacetic acid. Reactions were started after preincubation at 28 °C for 8 minutes by addition of the pterin cofactor (circles) or the amino acid substrate (diamonds); reactions contained 1 U μL^{-1} catalase and 0.05 U μL^{-1} SOD (filled) or were run in the absence of additives (open). Reactions were performed in duplicate.

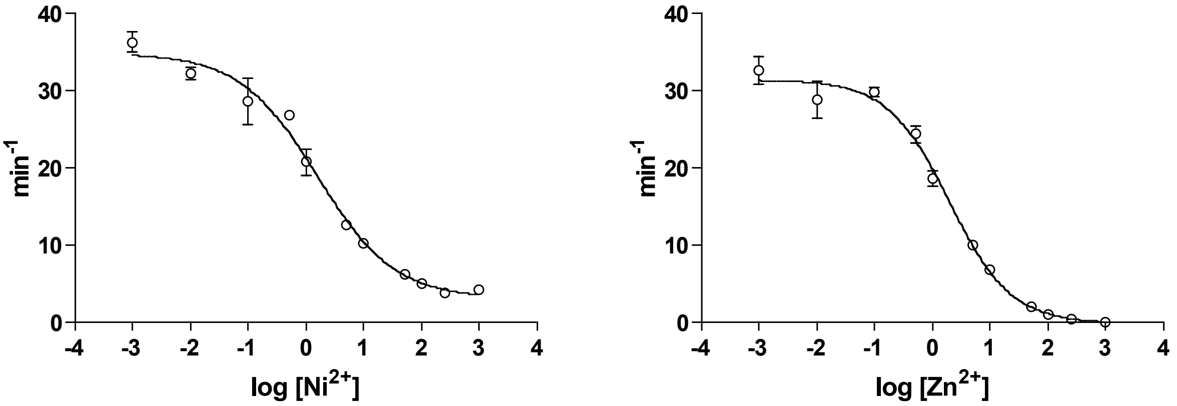

Entry	Inhibitor	K_i or IC50	IC50 95% confidence interval
1	Ni^{2+}	1.6 µM	1.0 – 2.4 µM
2	Zn^{2+}	2.0 µM	1.5 – 2.6 µM
3	Cu^{2+}	19 µM	11 – 31 µM

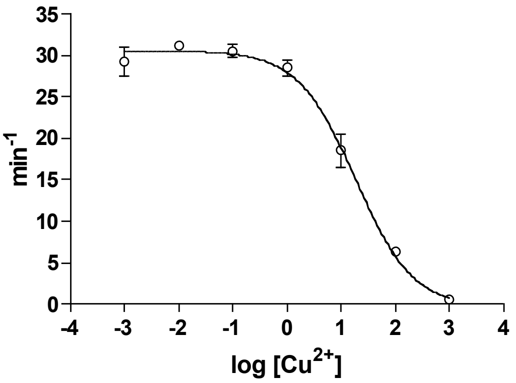

Figure S6. Phe3H with Ni^{2+}, Zn^{2+} and Cu^{2+} inhibition curves. Reaction conditions: 0.5 µM PhhA, 5 mM L-Phe, varying Me^{2+}; IC_{50} derived from non-linear regression to sigmoidal dose-response.

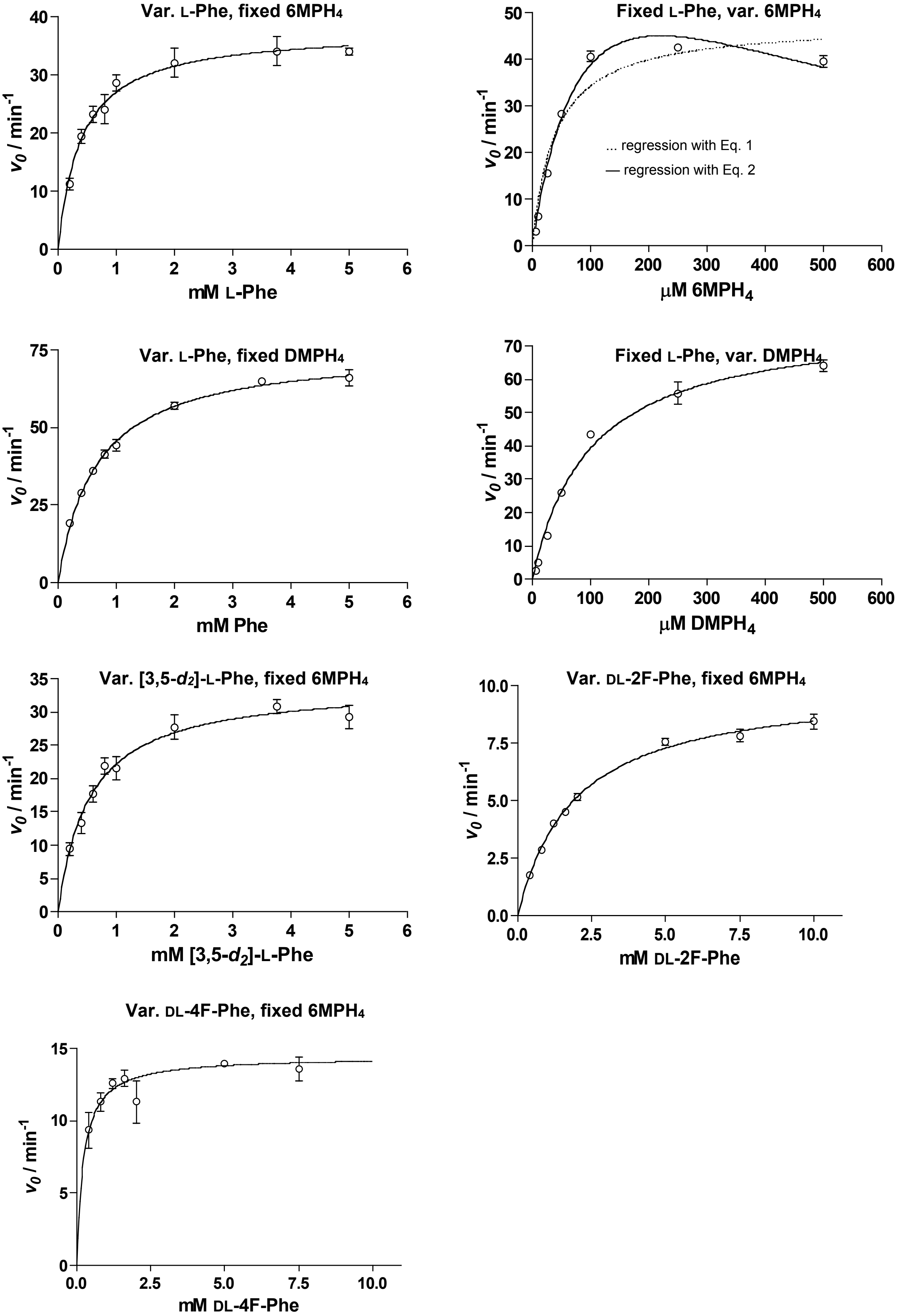

Figure S7. Steady-state (Michealis-Menten) kinetics of Phe3H catalysed reactions.

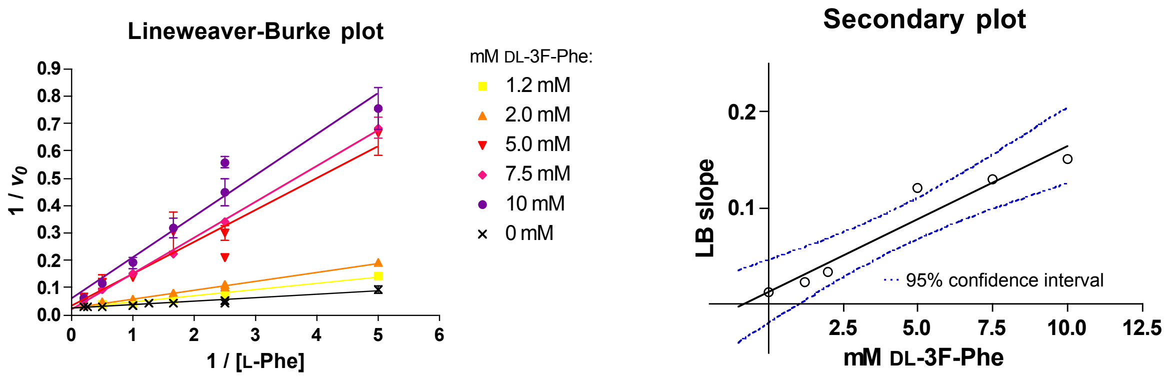

Figure S8. (a) Lineweaver-Burke plot of competitive inhibition of Phe3H by DL-3F-Phe. (b) Secondary plot of competitive inhibition of Phe3H by DL-3F-Phe.

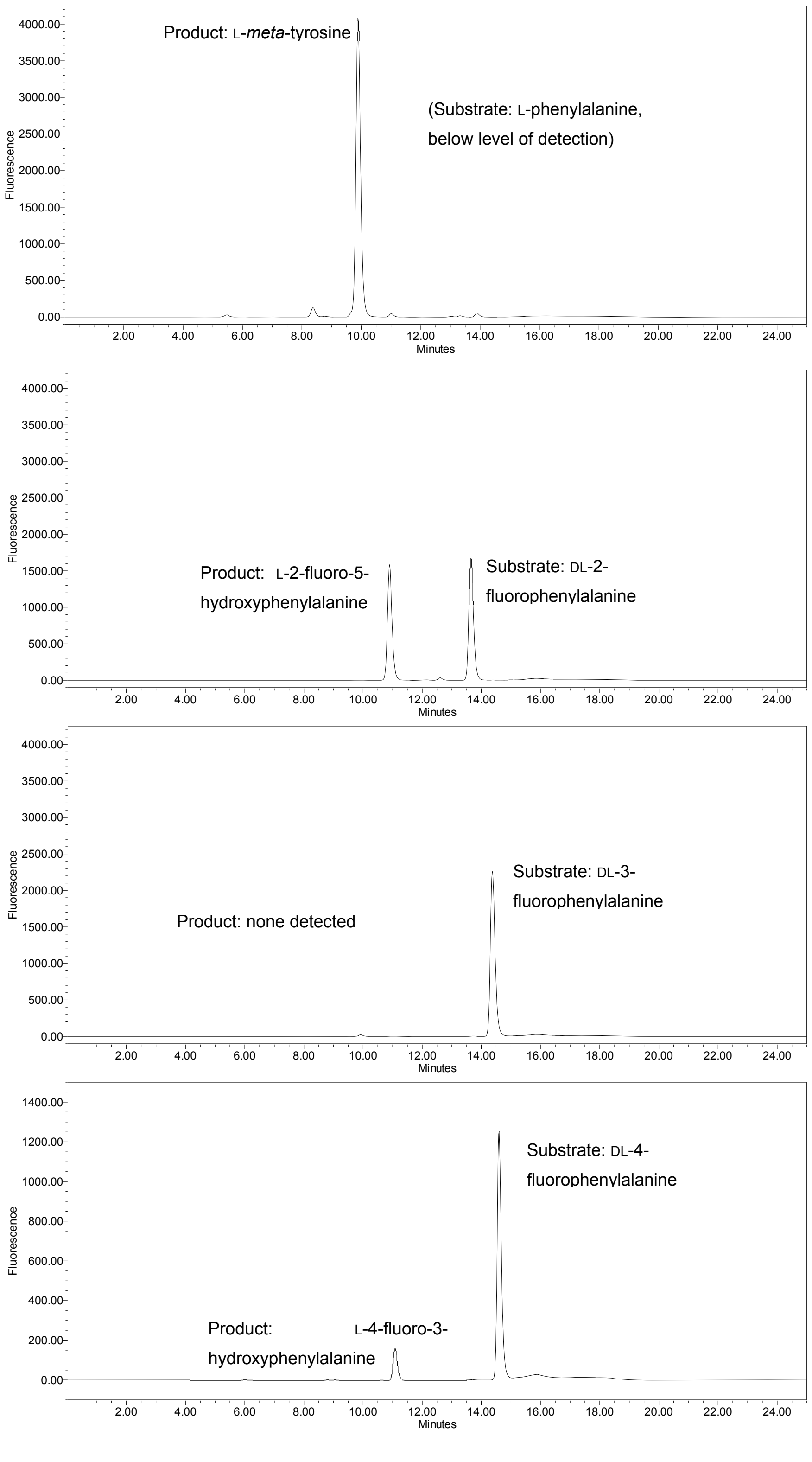

Figure S9. UPLC analysis of the substrate scope of Phe3H. Reaction mixtures were analysed on a Waters Alliance® HPLC equipped with a Waters 996 photodiode array detector and a 474 fluorescence detector. Compounds were separated on an Agilent Eclipse XDB-C8 column with 50 mM triethylamine : 60 mM trifluoroacetic acid, pH 2.0 as buffer A and methanol as solvent B (0 – 3 min 3% B, 3 – 11 min 3 – 30% B, 11 – 12 min 30 – 60% B, 12 – 15 min 60% B, 15 – 18 min 60 – 3% B, 18 – 23 min 3% B; flow rate 0.7 ml min^{-1}; λ_{ex} 270 nm, λ_{em} 305 nm).

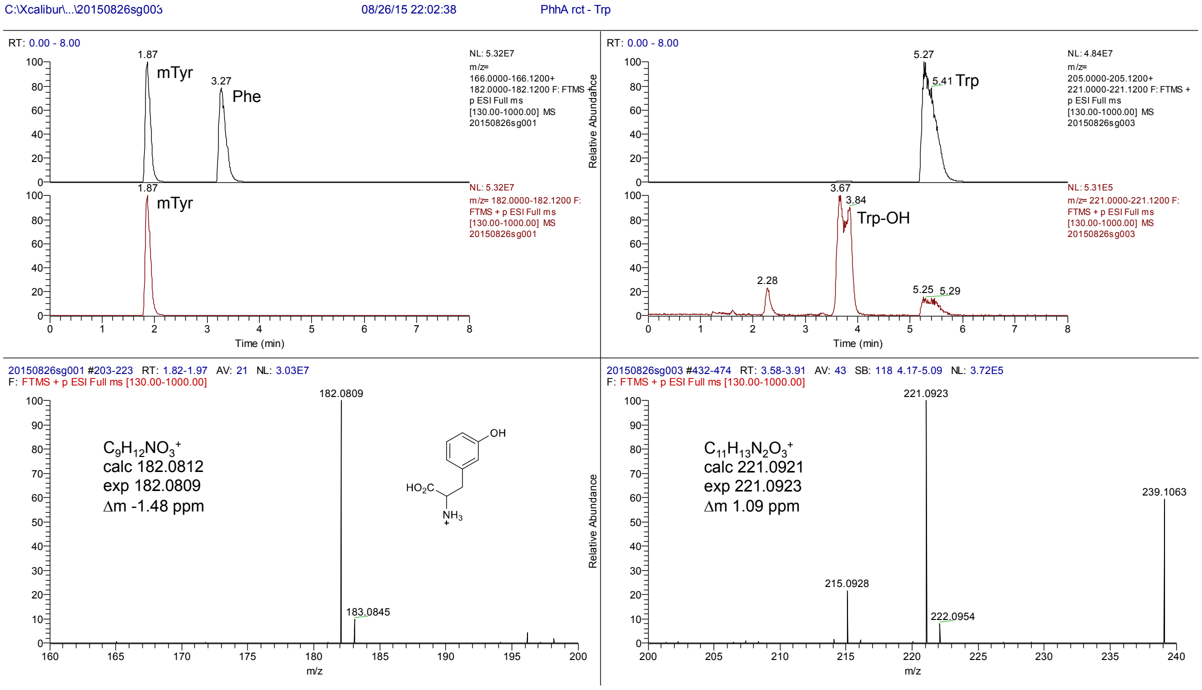

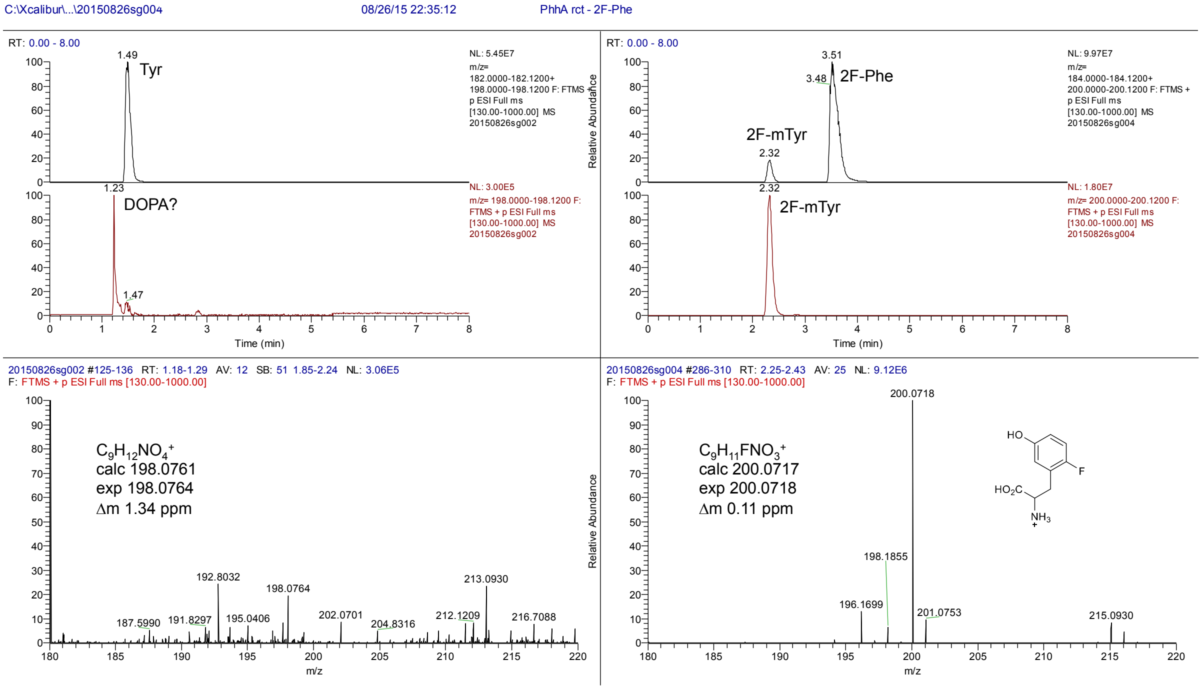

Figure S10. LC-MS analysis of the substrate scope of Phe3H.

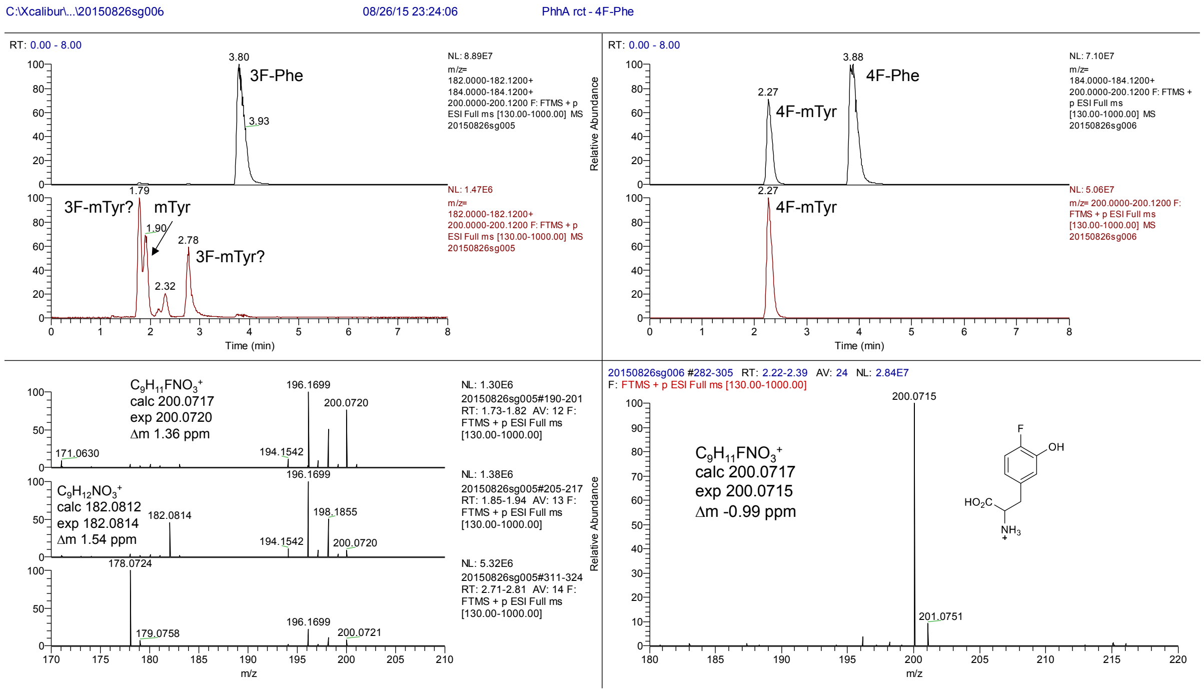

Figure S10. continued

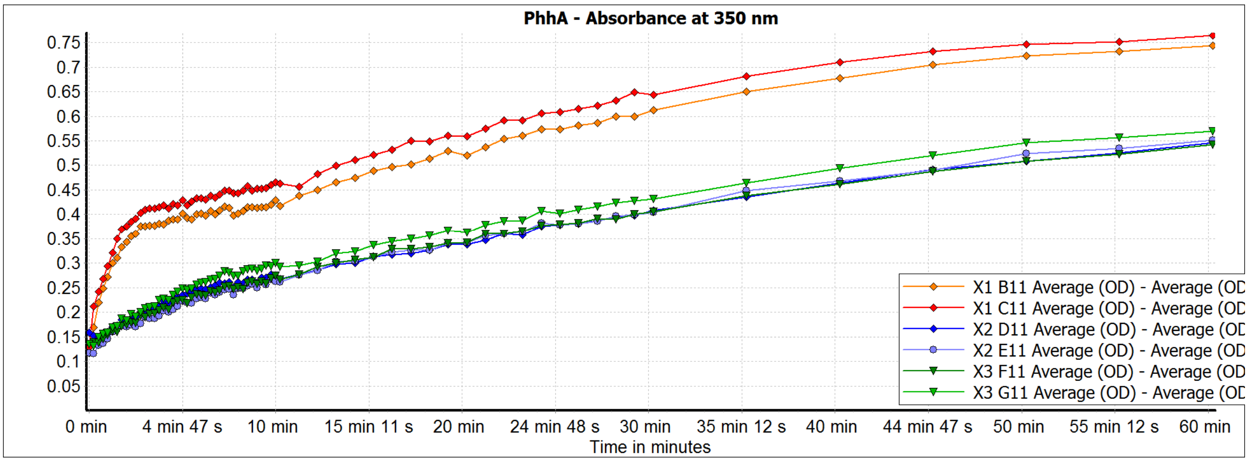

Figure S11. Absorbance spectroscopy of oxidation of $DMPH_4$ in the absence and presence of substrate or inhibitor. The reaction contained 1 µM Phe3H, 1 mM ascorbate, 0.05 U µL^{-1} superoxide dismutase, 5 U µL^{-1} catalase, 1 µM ferrous ammonium sulfate, 10 µM DTT, 0.5 mM $DMPH_4$, and 1.25 mM amino acid in 50 mM Tris-HCl, pH 8.0. Duplicate reactions are shown individually. X1 (gold, red) L-Phe; X2 (blue, purple) DL-3F-Phe; X3 (green) no amino acid.

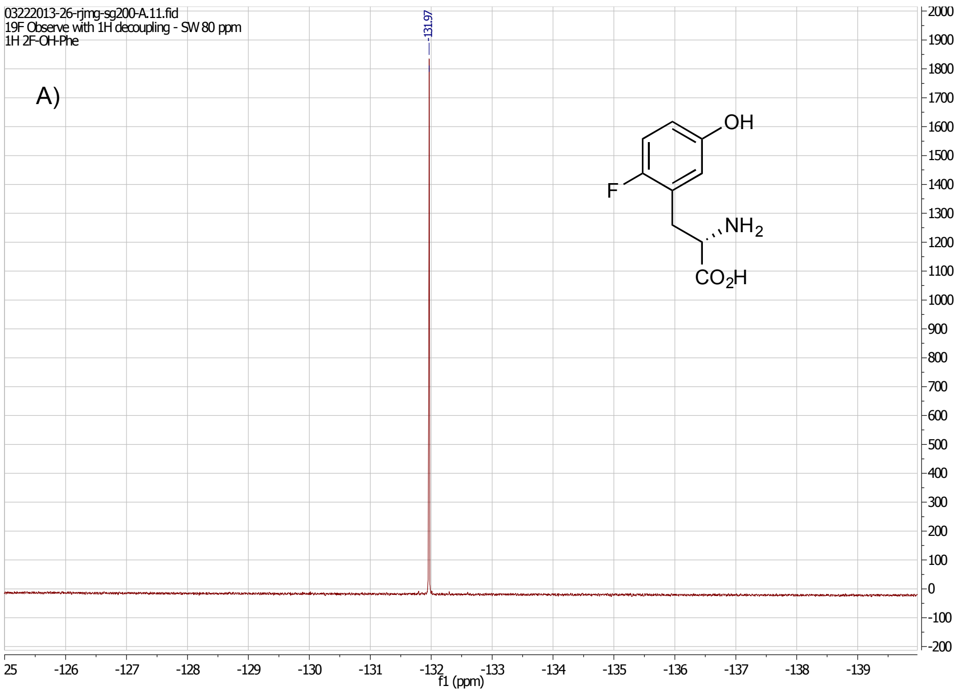

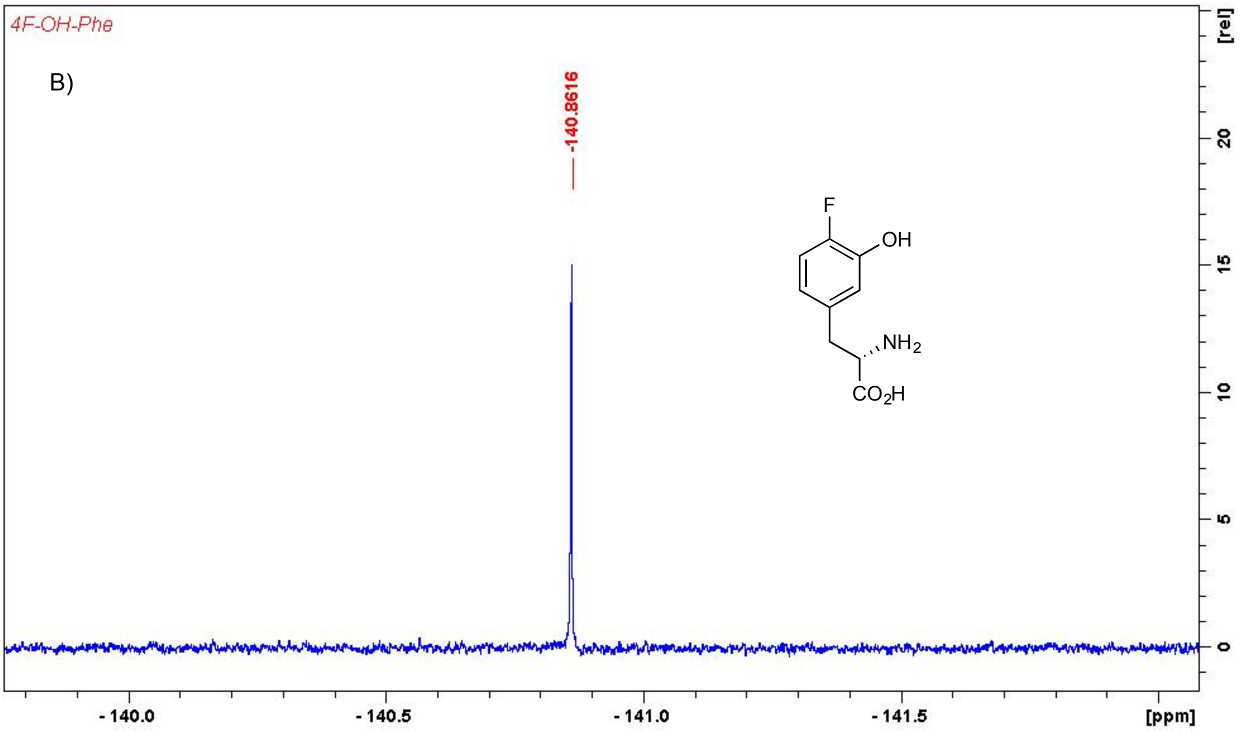

Figure S12. ^{19}F NMR (1H decoupled) of l-2-fluoro-5-hydroxyphenylalanine (A) and l-4-fluoro-3-hydroxy-phenylalanine (B).

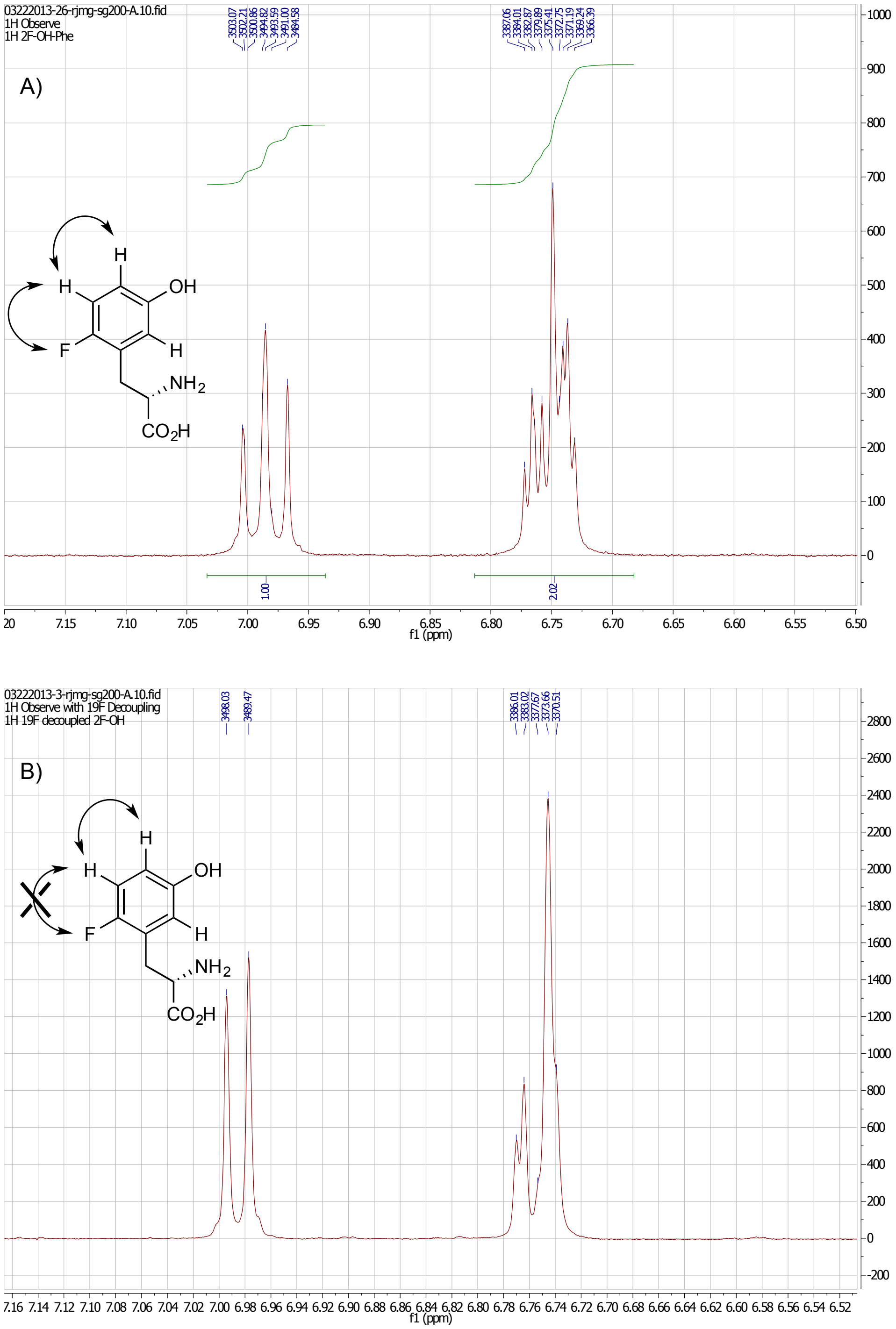

Figure S13. ^{1}H NMR (aromatic region) of l-2-fluoro-5-hydroxyphenylalanine in CD_3OD without ^{19}F decoupling (A) and ^{19}F decoupled (B).

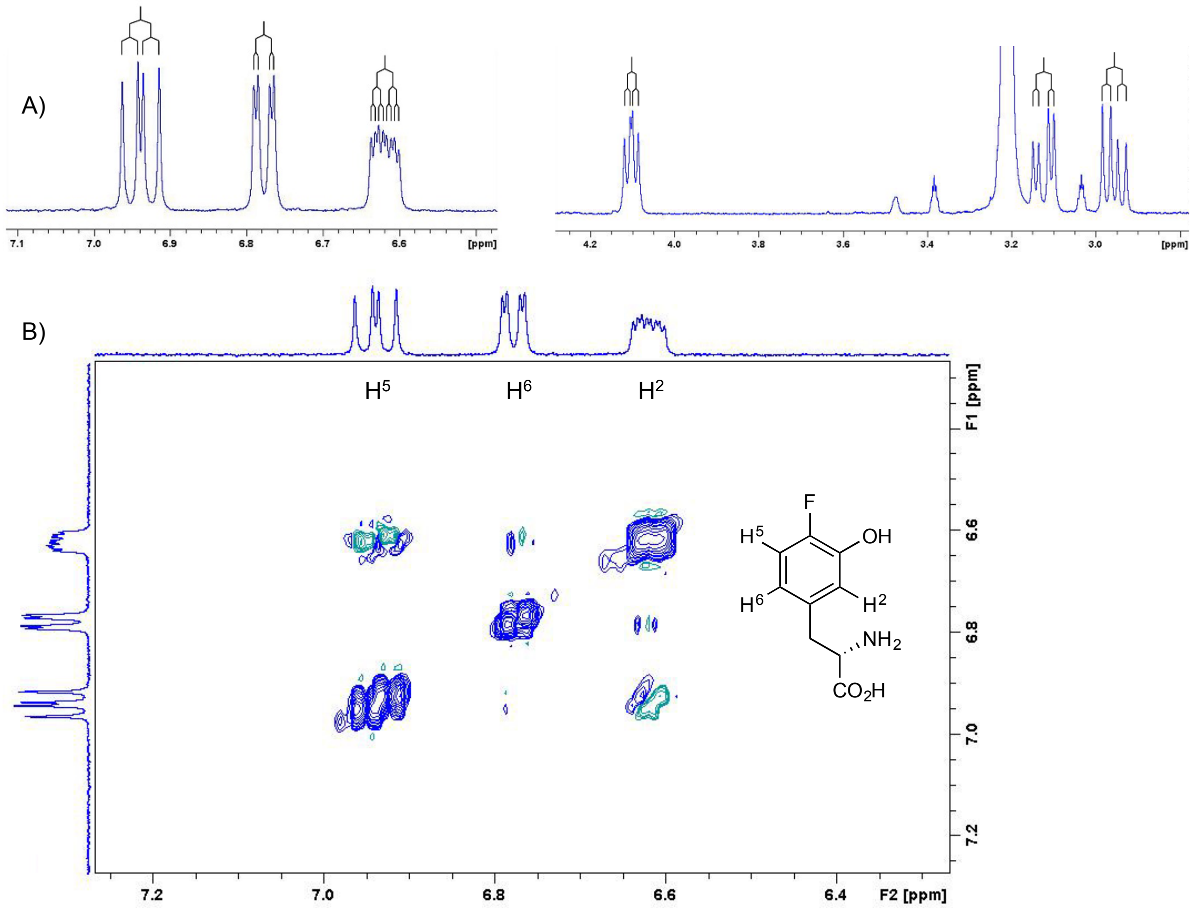

Figure S14. ^{1}H NMR (A) and NOESY (B) of l-4-fluoro-3-hydroxyphenylalanine in CD_3OD.

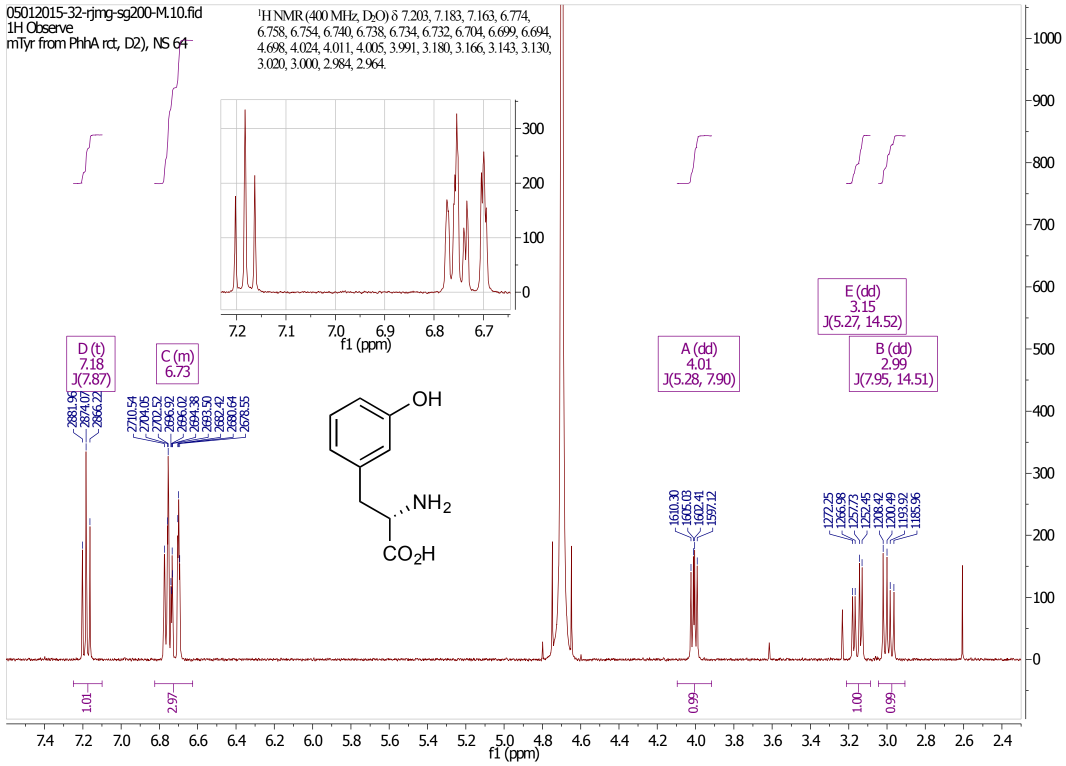

Figure S15. ^{1}H NMR of L-*meta*-tyrosine obtained from Phe3H reaction with L-Phe as substrate.

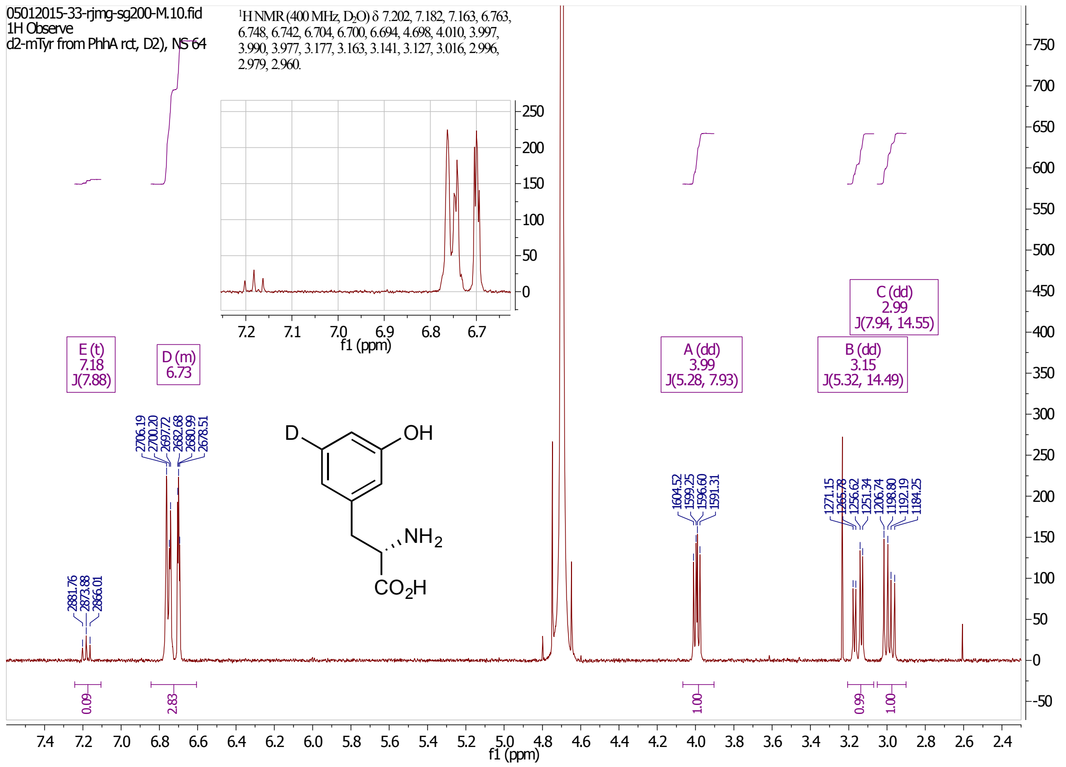

Figure S16. ^{1}H NMR of [d_1]-L-*meta*-tyrosine obtained from Phe3H reaction with L-[3,5-d_2]-Phe as substrate.

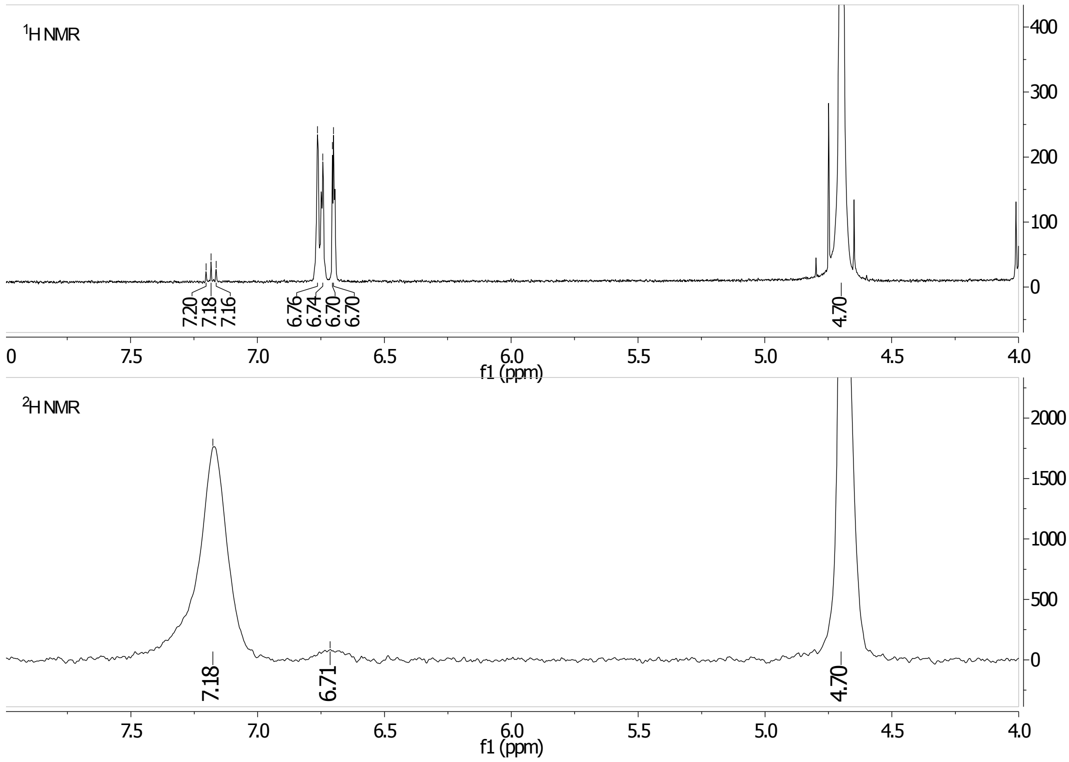

Figure S17. Comparison of ^1H and ^2H NMR spectra in D_2O and H_2O/D_2O, respectively, of L-*meta*-tyrosine obtained from Phe3H reaction with L-[3,5-d_2]-Phe as substrate (aromatic and solvent region). The deuterium signal at 6.71 ppm integrates to approximately 1% of the 7.18 ppm signal peak area.

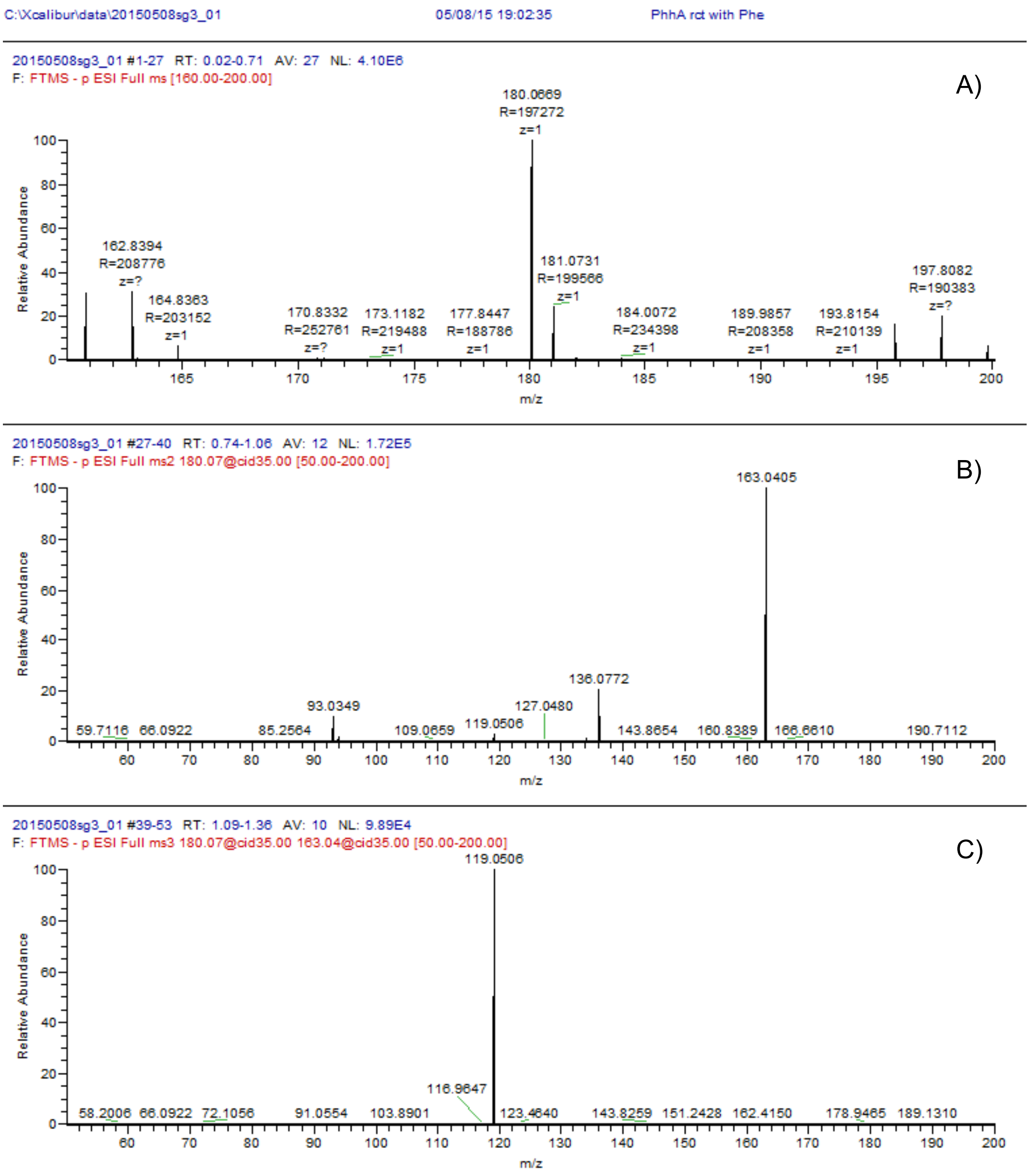

Figure S18. HRMS (ESI, -ve) analysis of L-*meta*-tyrosine obtained from Phe3H catalysed reaction with L-Phe as substrate. A) Full mass spectrum, calculated for $C_9H_{10}NO_3$ $[M-H]^-$ 180.0666, found 180.0669 (error 1.4 ppm); B) MS2 of molecular ion peak *m/z* 180.1 showing loss of NH_3 (δm 17); C) MS3 of *m/z* 180.1 → 163.0 showing subsequent loss of CO_2 (δm 44).

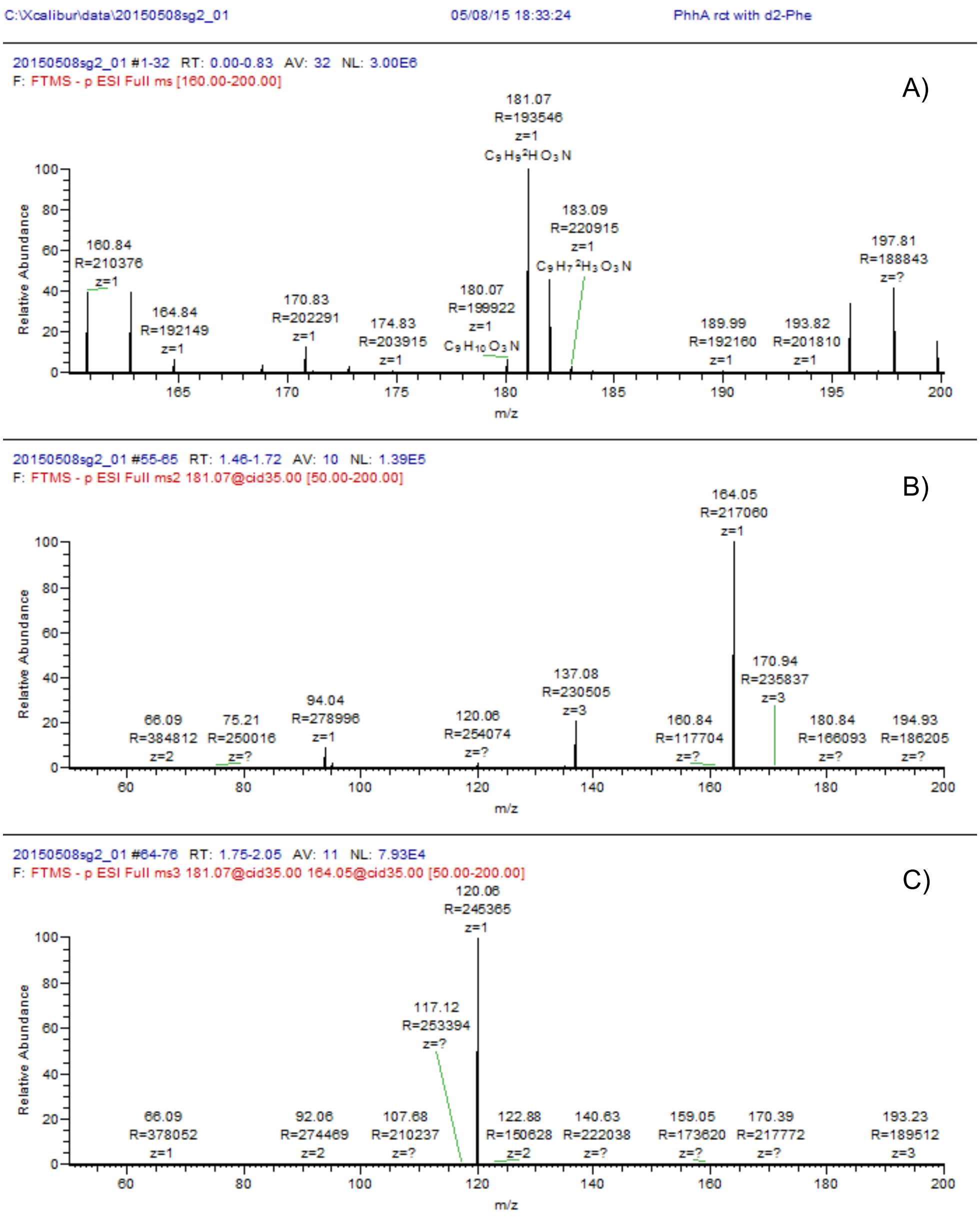

Figure S19. HRMS analysis (ESI, -ve) of [d_1]-L-*meta*-tyrosine obtained from Phe3H reaction with L-[3,5-d_2]-Phe as substrate. A) Full mass spectrum, calculated for $C_9H_9DNO_3$ $[M-H]^-$ 181.0729, found 181.0734 (error 2.7 ppm); B) MS2 of molecular ion peak *m/z* 181.1 showing loss of NH_3 (δm 17); C) MS3 of *m/z* 181.1 → 164.1 showing subsequent loss of CO_2 (δm 44).

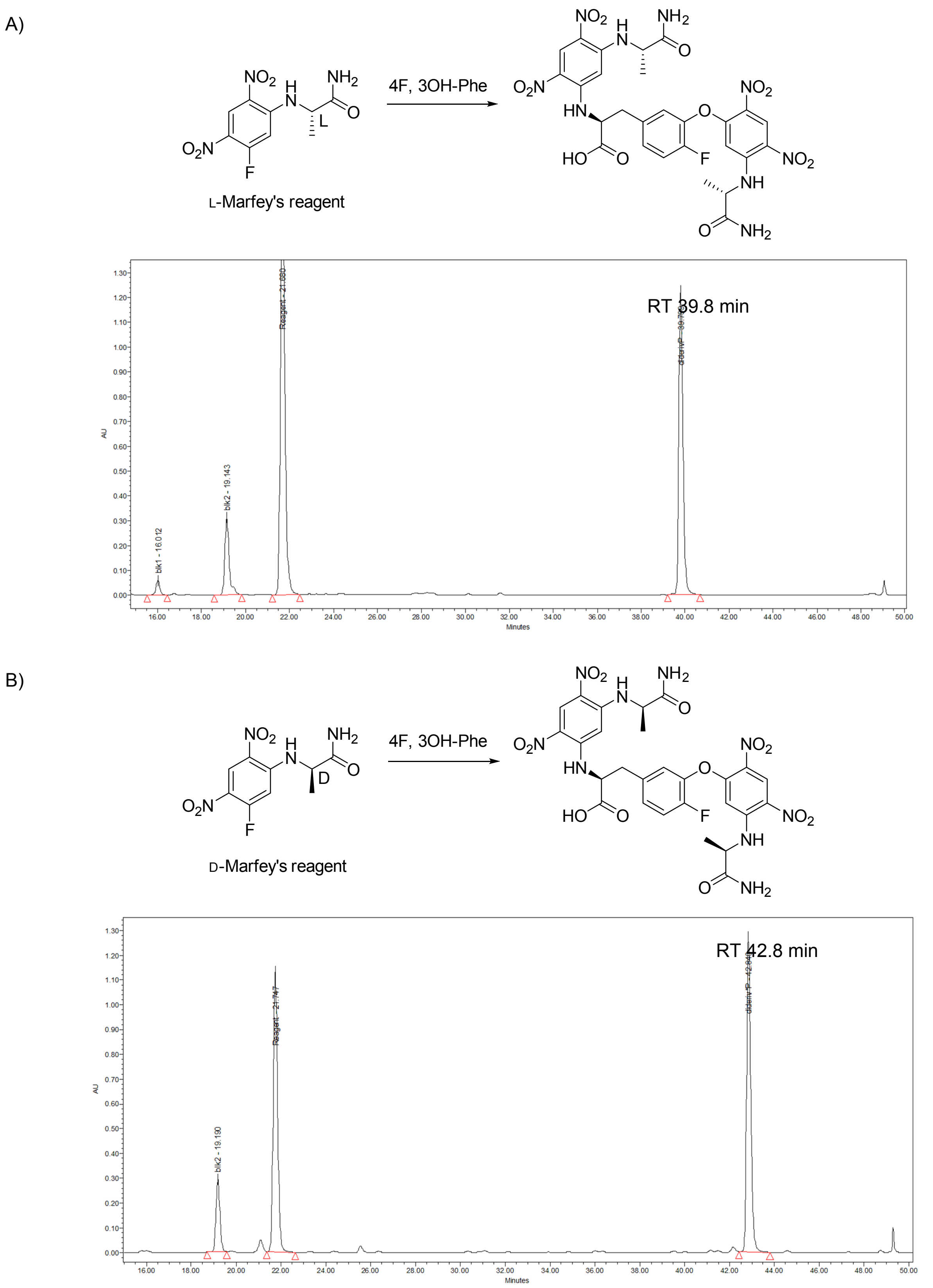

Figure S20. Determination of 4-fluoro-3-hydroxyphenylalanine stereochemistry using L-Marfey's reagent (A) and D-Marfey's reagent (B). Reactions conditions were chosen to form the diderivatised amino acid. A single peak in each case shows that a single enantiomer product has been formed.

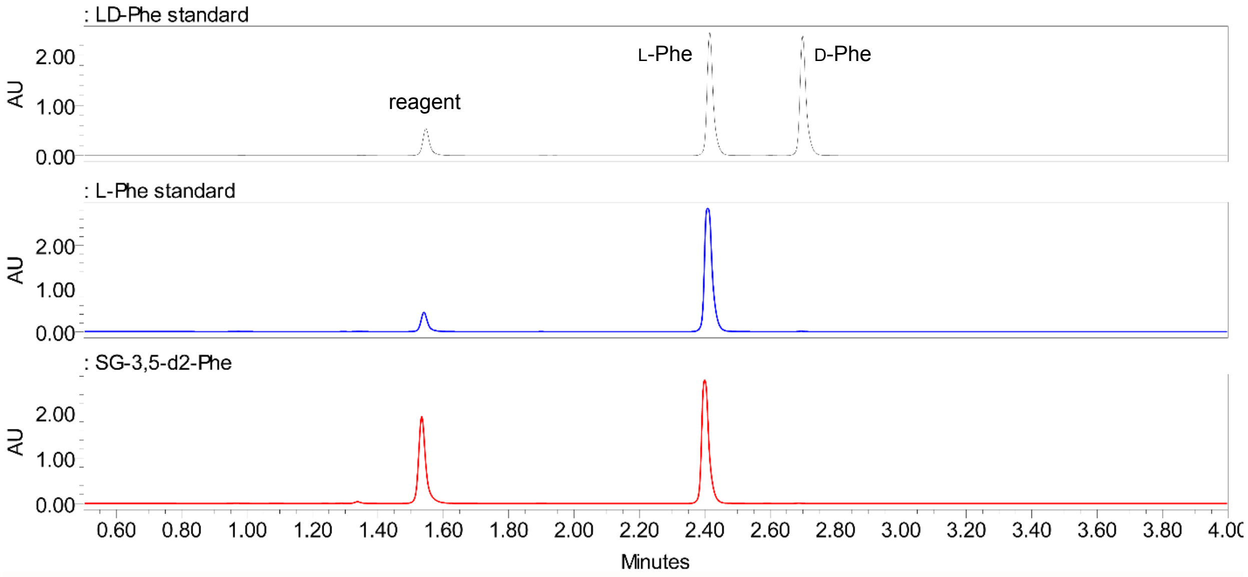

Figure S21. Verification of [3,5-d_2]-phenylalanine stereochemistry using Marfey's reagent.

```
Ph4H_CHRVO      ---------------------------------------------MNDRADFVVPDI-TT      14
SfaA            -----------------------------------------MEIGSGAPELTASSVYQQR      19
srosPhhA        --------------------------------------------MQRKHACITDAAYGQR      16
NpsK            --------------------------------------------MQRQHASITDAAYGQR      16
sclrPhhA        --------------------------------------------MQGPHAQMTDAAYEIR      16
SsaX            --------------------------------------------MQGHRDQVTDTAYEKR      16
PH4H_RAT        LRNDIGATVHELSRDKEKNTVPWFPRTIQELDRFANQILSYGAELDADHPGFKDPVYRAR      157
PH4H_HUMAN      LRHDIGATVHELSRDKKKDTVPWFPRTIQELDRFANQILSYGAELDADHPGFKDPVYRAR      157
TY3H_HUMAN      VR-----QVSEDVRSPAGPKVPWFPRKVSELDKCHHLVTKFDPDLDLDHPGFSDQVYRQR      233
TY3H_RAT        VR-----RVSDDVRSAREDKVPWFPRKVSELDKCHHLVTKFDPDLDLDHPGFSDQVYRQR      203


Ph4H_CHRVO      RKNVGLSHDANDFTLPQPLDRYSAEDHATWATLYQRQCKLLPGRACDEFMEGLERL----      70
SfaA            RDQIAASAAAYVPGEPIPEVEYTDAEHALWRLVSKRLADRHRHMAAPEFVEAAERL----      75
srosPhhA        RSEIGALSAGLTPDDPIPLVEYTDWEHEVWRLVCADLAVRHRVDAAAPYLQAAEEL----      72
NpsK            RSEIGALSAGLTPDDPIPLVEYTDWEHEVWRLVCADLAVRHRVDAAAPYLQAAEEL----      72
sclrPhhA        RSEIAALSTDLAPEDPIPVVEYTEWEHEVWRTVCVDLTARHRTDAAAEYLESAEQL----      72
SsaX            REEIAALSADVTLHGPVPVVEYVDWEHEVWRLACAALDARHRVDAAAEYLESAEAL----      72
PH4H_RAT        RKQFADIAYNYRHGQPIPRVEYTEEEKQTWGTVFRTLKALYKTHACYEHNHIFPLLEKYC      217
PH4H_HUMAN      RKQFADIAYNYRHGQPIPRVEYMEEEKKTWGTVFKTLKSLYKTHACYEYNHIFPLLEKYC      217
TY3H_HUMAN      RKLIAEIAFQYRHGDPIPRVEYTAEEIATWKEVYTTLKGLYATHACGEHLEAFALLERFS      293
TY3H_RAT        RKLIAEIAFQYKHGEPIPHVEYTAEEIATWKEVYVTLKGLYATHACREHLEGFQLLERYC      263
                *. ..          * *  .*   :   *              *.  . .    *

Ph4H_CHRVO      EVDADRVPDFNKLNQKLMAATGWKIVAVPGLIPDDVFFEHLANRRFPVTWWLREPHQLDY      130
SfaA            EVGGDGVPQLREVSDRLDQLTGFRLRPASGVVPFALFCGSLADGYFHSTQYLRDSATPFY      135
srosPhhA        AVPLDHVPQLRDVSARLASMSGFRLQSAAALVPLKEFCGSLANSVFRSTQYLRHPRSPLY      132
NpsK            AVPLDHVPQLRDVSARLASMSGFRLQSAAALVPLKEFCGSLANSVFRSTQYLRHPRSPLY      132
sclrPhhA        AVPLDHVPQLRDVSGRLGSISGFTFQSAPALVPLREFCGGLANSVFHSTQYLRHPRSPFY      132
SsaX            AIPLDHVPQLRDVSDRLTSISGFRLQSAATLVPLKEFCGSLANSVFRSTQYIRHPRAPLY      132
PH4H_RAT        GFREDNIPQLEDVSQFLQTCTGFRLRPVAGLLSSRDFLGGLAFRVFHCTQYIRHGSKPMY      277
PH4H_HUMAN      GFHEDNIPQLEDVSQFLQTCTGFRLRPVAGLLSSRDFLGGLAFRVFHCTQYIRHGSKPMY      277
TY3H_HUMAN      GYREDNIPQLEDVSRFLKERTGFQLRPVAGLLSARDFLASLAFRVFQCTQYIRHASSPMH      353
TY3H_RAT        GYREDSIPQLEDVSRFLKERTGFQLRPVAGLLSARDFLASLAFRVFQCTQYIRHASSPMH      323
                    * :*::..:.  *   :*: :  .  ::    *   **   *  * ::*.     :
                                                                      C187
Ph4H_CHRVO      LQEPDVFHDLFGHVPLLINPVFADYLEAYGKGGVKAKALGALPMLARLYWYTVEFGLINT      190
SfaA            STEPDILHEVIGHGSALADDRFANLYRLAGEAVRRVESEDAVQFVAKTFWFTLECGLLDA      195
srosPhhA        SEDPDMLHDLVGHGIALANARFAHLYRLAGEAAARVQSADALQFIGKVFWFTLECGVVRE      192
NpsK            SEDPDMLHDLVGHGIALANTRFAHLYRLAGEAAARVQSADALQFIGKVFWFTLECGVVRE      192
sclrPhhA        TEDPDLLHDLVGHGNVLASDRFARLYRLAGNAAARVHSTEALQFIGKVFWFTLECGVVRE      192
SsaX            SEDPDMLHDLVGHGNVLANDRFVRLYRLAGEAANRVESDEALQFIGKVFWFTLECGVVRE      192
PH4H_RAT        TPEPDICHELLGHVPLFSDRSFAQFSQEIGLASL-GAPDEYIEKLATIYWFTVEFGLCKE      336
PH4H_HUMAN      TPEPDICHELLGHVPLFSDRSFAQFSQEIGLASL-GAPDEYIEKLATIYWFTVEFGLCKQ      336
TY3H_HUMAN      SPEPDCCHELLGHVPMLADRTFAQFSQDIGLASL-GASDEEIEKLSTLYWFTVEFGLCKQ      412
TY3H_RAT        SPEPDCCHELLGHVPMLADRTFAQFSQDIGLASL-GASDEEIEKLSTVYWFTVEFGLCKQ      382
                  :**  *::.**   : .  *.   .  * .         :  :.  :*:*:* *:
                         T202
Ph4H_CHRVO      PAGMRIYGAGILSSKSESIYCLDSASPNRVGFDLMRIMNTRYRIDTFQKTYFVIDSFKQL      250
SfaA            ADGPRAYGASVVSSYGELEHFRS-A--EIRPLDIADMAHVDYDITQYQTTFYSARSLTHL      252
srosPhhA        RGERKAYGATLVSSYGELDHFRS-A--AFLPLDIESLVDIEYDISTYQPLLFEADSLDQV      249
NpsK            HGERKAYGATLVSSYGELDHFRS-A--AFLPLDIESLVDIEYDISTYQPLLFEADSLDQV      249
sclrPhhA        RGERKAYGATLVSSYGELDHFRS-A--DFRPLDIKSLADVEYDISTYQPILFEADSMDEV      249
SsaX            RGERKAYGATLVSSYGELDHFRS-A--EFRPLSIESLVDVKYDITTYQPLLFEADSMNEV      249
PH4H_RAT        GDSIKAYGAGLLSSFGELQYCLS-DKPKLLPLELEKTACQEYSVTEFQPLYYVAESFSDA      395
PH4H_HUMAN      GDSIKAYGAGLLSSFGELQYCLS-EKPKLLPLELEKTAIQNYTVTEFQPLYYVAESFNDA      395
TY3H_HUMAN      NGEVKAYGAGLLSSYGELLHCLS-EEPEIRAFDPEAAAVQPYQDQTYQSVYFVSESFSDA      471
TY3H_RAT        NGELKAYGAGLLSSYGELLHSLS-EEPEVRAFDPDTAAVQPYQDQTYQPVYFVSESFNDA      441
                    : *** ::** .*  :  .        :.        *    :*   :   *: .

Ph4H_CHRVO      FDATAPDFAPLYL------QLADAQPWGAGDVAPDDLVLNAGDRQGWADTEDV-------      297
SfaA            EDVAGEFWASCDDTSIEKLMAVDI------------------------------------      276
srosPhhA        EDVLGAFWGSCD------------------------------------------------      261
NpsK            EDVLGAFWGSCD------------------------------------------------      261
sclrPhhA        EDTVGSFWDTCDDDSIAALLGGTSRSVTPH------------------------------      279
SsaX            EDLVGSFWDTCDDDSIAALFSGASRSVGTRR-----------------------------      280
PH4H_RAT        KEKVRTFAATIPRP-----FSVRYDPYTQRVEVLDNTQQLKILADSINSEVGILCNALQK      450
PH4H_HUMAN      KEKVRNFAATIPRP-----FSVRYDPYTQRIEVLDNTQQLKILADSINSEIGILCSALQK      450
TY3H_HUMAN      KDKLRSYASRIQRP-----FSVKFDPYTLAIDVLDSPQAVRRSLEGVQDELDTLAHALSA      526
TY3H_RAT        KDKLRNYASRIQRP-----FSVKFDPYTLAIDVLDSPHTIQRSLEGVQDELHTLAHALSA      496
                 :
```

Figure S22. Multiple sequence alignment of Phe4H from *Chromobacterium violaceum* with phenylalanine 3-hydroxylases from the pacidamycin, napsamycin, sansanmycin and sanglifehrin pathways, a hypothetical protein from *Streptomyces filamentosus NRRL 15998,* rat and human Phe4H and tyrosine *meta*-hydroxylase.

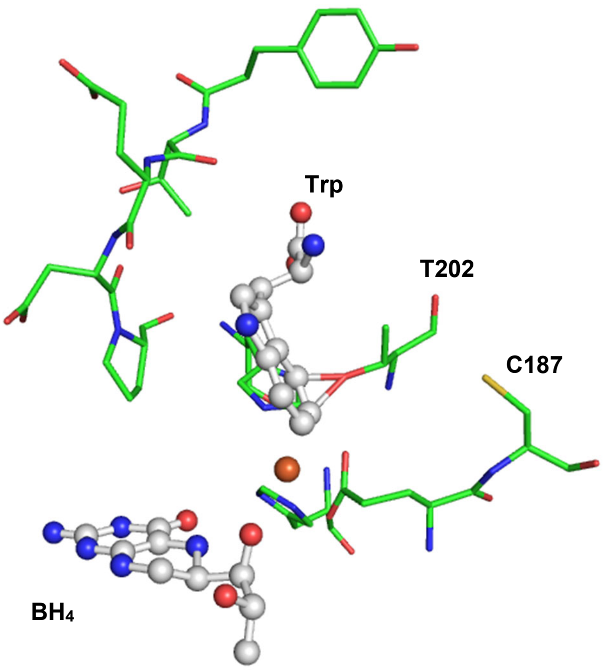

Figure S23. Model of Phe3H with Trp and BH_4 in the active site showing the position of putative base residues C187 and T202. The model was created using l-TASSER.[12–14]

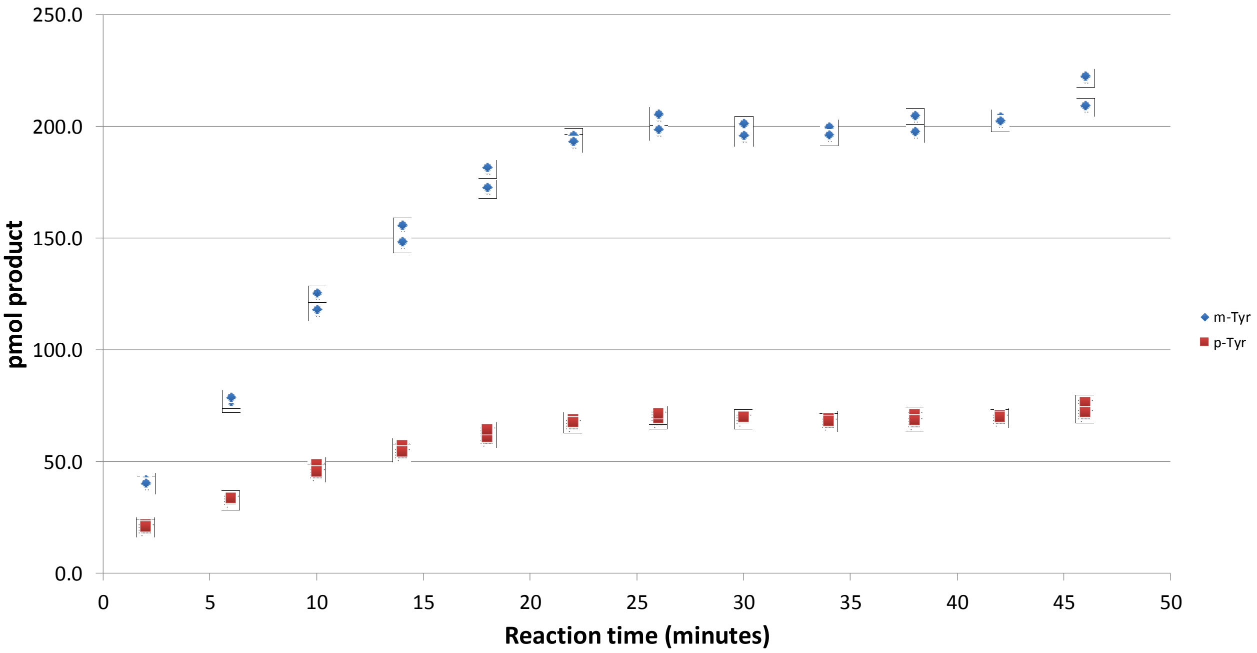

Figure S24. Time course of *m*Tyr and *p*Tyr formation by Phe3H .

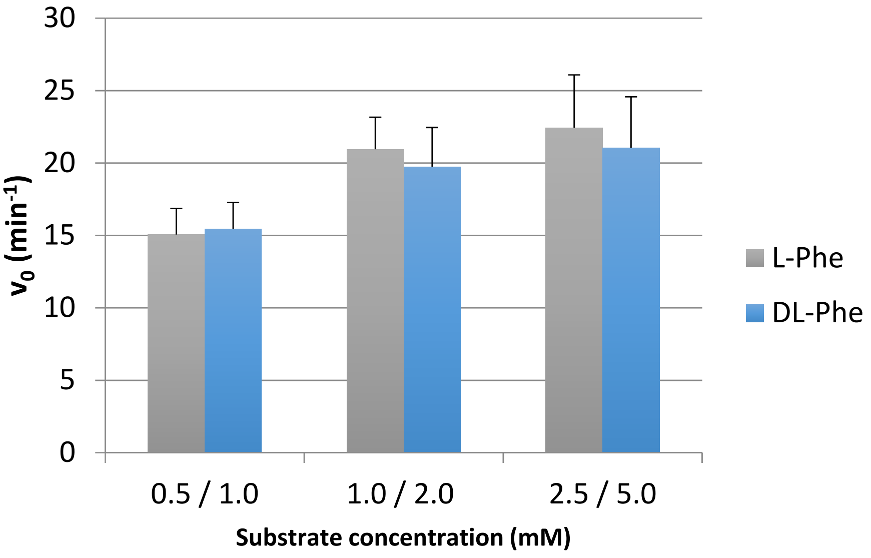

Figure S25. Comparison of rate of velocity of Phe3H catalysed mTyr formation with L-Phe (grey bars) and DL-Phe (blue bars). Double the concentration of DL-Phe was used, such that the effective concentration of L-Phe was kept constant.

Table S1. Conversion of L-Phe (25 mM) to Tyr or mTyr in 3 hours reaction time at 28 °C.

	Total conversion
**WT**	31
**C187S**	3
**C187A**	10
**T202A**	7
**T202S**	17

3 References

1 W. Zhang, B. D. Ames and C. T. Walsh, Identification of Phenylalanine 3-Hydroxylase for meta-Tyrosine Biosynthesis, *Biochemistry*, 2011, **50**, 5401–5403.

2 J. J. A. Marota and R. Shiman, Stoichiometric reduction of phenylalanine hydroxylase by its cofactor: a requirement for enzymatic activity, *Biochemistry*, 1984, **23**, 1303–1311.

3 A. Martinez, K. K. Andersson, J. Haavik and T. Flatmark, EPR and 1H-NMR spectroscopic studies on the paramagnetic iron at the active site of phenylalanine hydroxylase and its interaction with substrates and inhibitors, *Eur. J. Biochem.*, 1991, **198**, 675–682.

4 A. Martinez, J. Haavik and T. Flatmark, Cooperative homotropic interaction of l-noradrenaline with the catalytic site of phenylalanine 4-monooxygenase, *Eur. J. Biochem.*, 1990, **193**, 211–219.

5 C. Kitatsuji, K. Izumi, S. Nambu, M. Kurogochi, T. Uchida, S. Nishimura, K. Iwai, M. R. O'Brian, M. Ikeda-Saito and K. Ishimori, Protein oxidation mediated by heme-induced active site conversion specific for heme-regulated transcription factor, iron response regulator., *Sci. Rep.*, 2016, **6**, 18703.

6 Y. M. Báez-Santos, A. M. Mielech, X. Deng, S. Baker and A. D. Mesecar, Catalytic Function and Substrate Specificity of the Papain-Like Protease Domain of nsp3 from the Middle East Respiratory Syndrome Coronavirus, *J. Virol.*, 2014, **88**, 12511–12527.

7 J. R. Clasman, Y. M. Bez-Santos, R. C. Mettelman, A. O'Brien, S. C. Baker and A. D. Mesecar, X-ray structure and enzymatic activity profile of a core papain-like protease of MERS coronavirus with utility for structure-based drug design, *Sci. Rep.*, 2017, **7**, 40292–40305.
